# Visible-Light-Active BiOI/TiO_2_ Heterojunction Photocatalysts for Remediation of Crude Oil-Contaminated Water

**DOI:** 10.1021/acsomega.3c04359

**Published:** 2023-11-07

**Authors:** Blessing Ogoh-Orch, Patricia Keating, Aruna Ivaturi

**Affiliations:** Smart Materials Research and Device Technology (SMaRDT) Group, Department of Pure and Applied Chemistry, Thomas Graham Building, University of Strathclyde, 295 Cathedral Street, Glasgow G1 1XL, UK

## Abstract

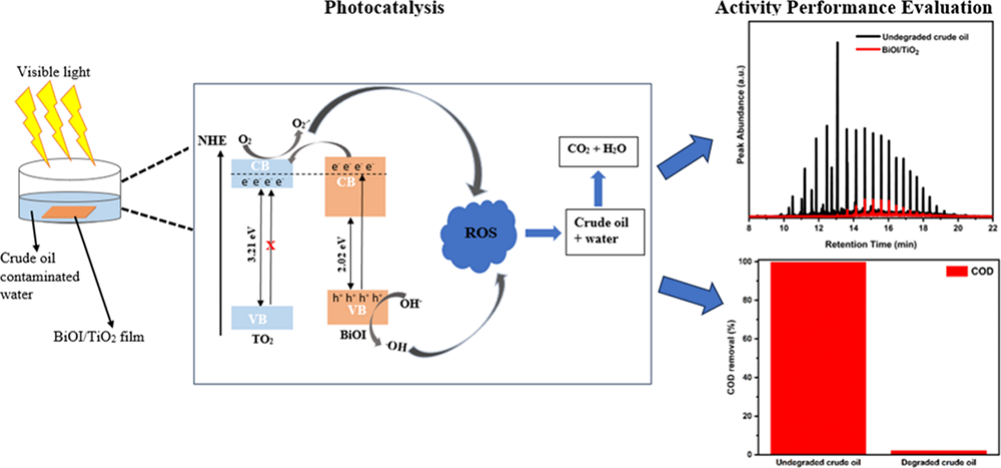

In this study, BiOI-sensitized TiO_2_ (BiOI/TiO_2_) nanocomposites with different levels of BiOI deposited via sequential ionic layer adsorption and reaction (SILAR) have been explored for the degradation of methyl orange, 4-chlorophenol (4-CP), and crude oil in water under visible (>400 nm) irradiation with excellent degradation performance. The reaction progress for methyl orange and 4-chlorophenol was monitored by a UV–vis spectrophotometer, and the degradation of the crude oil hydrocarbons was determined by GC-MS. The BiOI/TiO_2_ heterojunction improves separation of photogenerated charges, which enhances the degradation efficiency. Evaluation of the visible-light photocatalytic performance of the synthesized catalysts against methyl orange degradation confirmed that four SILAR cycles are the optimal deposition condition for the best degradation efficiency. The efficiency was further confirmed by degrading 4-CP and crude oil, achieving 38.30 and 85.62% degradation, respectively, compared with 0.0% (4-CP) and 70.56% (crude oil) achieved by TiO_2_. The efficiency of TiO_2_ in degrading crude oil was mainly due to adsorption along with photolysis. This study provides a simple and cost-effective alternative to traditional remediation methods requiring high energy consumption for remediation of crude oil-polluted water and refinery wastewater using visible-light photocatalysis along with adsorption.

## 1 Introduction

Water pollution resulting from a crude oil spill is a major environmental concern. Oil spills occur through neglect, oil bunkering, pipeline corrosion, or accidental spill or by inadequate treatment and discharge of petroleum effluents.^1^ The increasing energy demand has made crude oil processing an important issue. Crude oil plays important roles in the society ranging from being a raw material for numerous consumer goods to being a major source of revenue for countries such as USA, Russia, Nigeria, and Saudi Arabia.^2,3^ However, its spill in the marine environment has many ill effects such as destruction of marine shorelines used for tourist sites and recreational centers, health, food, etc., thereby lessening the importance of the oil industry to the economy. Research on petroleum hydrocarbon degradation show that polycyclic aromatic hydrocarbons (PAH) and high molecular weight alkanes are resistant to biodegradation; therefore, photodegradation is very important in promoting the bioavailability and degradation of these recalcitrant compounds.^4^ Research on photodegradation of crude oil pollutes is being carried out by various groups, since the first study by Hansen et al. dated back to 1975.^3,5−10^

The fundamental element in weathering processes in the marine environment, where hydrocarbons are converted to water-soluble and tiny mobile molecules, is the photo-oxidation of petroleum contents that have been spilt. Studies focus on the photocatalytic conversion of hydrocarbons and potential uses of photocatalysis to clean up maritime oil spills.^11−14^

Photocatalysis has attracted increasing interest as a technique for destroying organic pollutants in water.^8,10,15^ The proposed materials for photocatalysis are mostly the semiconductors, where the photogenerated holes and electrons act as strong oxidizing and reducing agents, respectively. Among these semiconductors, titanium dioxide (anatase) has been greatly used due to its outstanding properties such as nontoxicity, chemical and biological inertness, photostability, biocompatibility, low cost, and resistance to chemical and photo corrosion.^16^ Unfortunately, TiO_2_ can only absorb in the UV region due to its wide energy band gap (3.2 eV), thereby limiting its applications as only 5–8% of UV light from the solar spectrum is being utilized. In contrast, visible light (400–700 nm) is abundant (46% of the solar spectrum). Also, TiO_2_ suffers from fast charge recombination.^17^ Moreover, for effluent treatment, photocatalytic applications are usually studied using powdered (suspension) catalysts, which are difficult to recover from the solution and reuse. The potential use of solar irradiation of photocatalysts in future technology application for water pollution remediation in areas lacking electricity infrastructure, which restricts the usage of traditional water treatment systems, is appealing. Therefore, inclusive of the outstanding properties of TiO_2_ mentioned above, for a successful photocatalytic application, the photocatalyst should be visible light active and easy to reclaim and reuse. Much research has been carried out to address these limitations via introduction of dopants, surface sensitization with carbon-based nanomaterials, and formation of heterojunctions.^3,8,9,18−20^

The formation of heterojunctions is one of the promising ways of improving visible-light activity and addressing charge recombination issues of TiO_2_. Usually, a heterojunction comprises the main semiconductor (TiO_2_) with a wide band gap in contrast with a narrow-band-gap semiconductor sensitizer. The presence of the sensitizer allows the composite to absorb visible light from the solar spectrum causing the excitation of electrons from the photocatalyst surface. The transfer of the electron/hole (e^–^/h^+^) pair between the two semiconductors reduces charge recombination, thereby increasing interfacial charge transfer compared to single semiconductors.^21^

Recently, bismuth-based compounds such as bismuth oxyhalides (BiOF, BiOCl, BiOBr, and BiOl) have drawn great attention due to their ability to photocatalytically degrade organic pollutants owing to their excellent optical and electrical properties.^22−25^ Therefore, a heterojunction between TiO_2_ and bismuth-based compounds with a narrow band gap such as BiOI is an ideal choice. BiOI is well known for its photocatalytic degradation of pollutants under visible-light illumination due to its narrow band gap (approximately 1.73–2.1 eV)^26−28^ and simple electronic structure, and because it is a p-type semiconductor, it can form a p–n junction with n-type semiconductors such as Bi_2_WO_6_, TiO_2_, g-C_3_N_4_, WO_3_, CdS, and Fe_3_O_4_,^18,27−33^ thereby improving the photoactivity of the photocatalyst.

In this study, a BiOI/TiO_2_ heterojunction photocatalyst with different-level deposition of bismuth oxyiodide was prepared via the sequential ionic layer adsorption and reaction (SILAR) method on doctor-bladed TiO_2_ mesoporous layers coated on FTO substrates. The photodegradation activity of BiOI/TiO_2_ has been widely explored especially for dyes^18,26−28,34−38^ and once for 4-chlorophenol^18^ degradation under visible light. The use of dyes as model compounds for photocatalytic degradation has been considered not ideal due to their visible-light-absorbing nature, which can photosensitize semiconductors^21^ and as such may not substantiate the intrinsic photocatalytic activity of the photocatalyst. This work focuses on the photocatalytic degradation of Nigerian crude oil (Bonny light from Bonny city, Rivers State, Nigeria)-contaminated water using BiOI/TiO_2_ under visible-light irradiation. Chemical adsorption using activated carbon is the usual treatment method for crude oil wastewater remediation in the oil industries. However, adsorption only removes the pollutants but does not degrade them and it is difficult to regenerate used carbon, which is usually disposed by incineration (which is not an environmentally friendly approach).^21^ Many studies on photocatalytic degradation of crude oil pollutants in water have been carried out,^39−43^ but to the best of our knowledge, this is the first time BiOI/TiO_2_ has been used to degrade crude oil hydrocarbons in water.

## 2 Experimental Section

### 2.1 Materials and Chemicals

TiO_2_ paste was purchased from Greatcell Solar [18NR-AO, a blend of active anatase (20 nm) and a larger anatase (up to 450 nm)]. Bi(NO_3_)_3_·5H_2_O (>98%), KI (>99%), methyl orange (>95%), 4-chlorophenol (4-CP, >99%), dichloromethane (DCM), chloroform, poly aromatic hydrocarbon standards, and alkane standards (C_7_–C_40_) were obtained from Sigma-Aldrich and used without further purification. The crude oil sample was obtained from Bonny city, Rivers State, Nigeria. FTO (fluorine tin oxide, TEC 7, 2.2 mm, 8 Ω/sq) glass substrates were supplied by NSG Pilkington.

### 2.2 Preparation of TiO_2_ Films

TiO_2_ paste was deposited onto FTO glass substrates through the doctor blading method using a 3M Scotch tape as spacer. Before the deposition, FTO glass was cut into 0.8 cm × 3.5 cm (for methyl orange and 4-CP degradation), 1 cm × 2 cm (for SEM and EIS analyses), 2.5 cm × 2.5 cm for XPS analysis, 3 cm × 3 cm (for crude oil degradation), and 2.5 cm × 2.5 cm microscope slide for XRD and DRS analyses using a glass cutter. The cut FTO glass and microscope slide substrates were cleaned by washing with 2% Hellmanex solution, brushed, and rinsed with tap water and then with DI water. The substrates were further cleaned by sequential sonication using DI water, isopropyl alcohol (IPA), and acetone for 15 min each. The substrates were then dried using compressed air, followed by oxygen plasma cleaning prior to TiO_2_ paste deposition. After deposition, the TiO_2_ film-coated substrates were allowed to stand for 10 min before sintering. The substrates with the TiO_2_-coated conductive side up were placed on a hot plate set at 120 °C for 10 min, after which they were transferred to a programmable hot plate and heated through 125 °C for 5 min, 325 °C for 5 min, 375 °C for 5 min, and 450 °C for 30 min to remove the organic binders, resulting in highly porous titania films, which were allowed to cool to room temperature before removing.

### 2.3 Sensitization of TiO_2_ Films with Bismuth Oxyiodide (BiOI)

**Figure 1:**
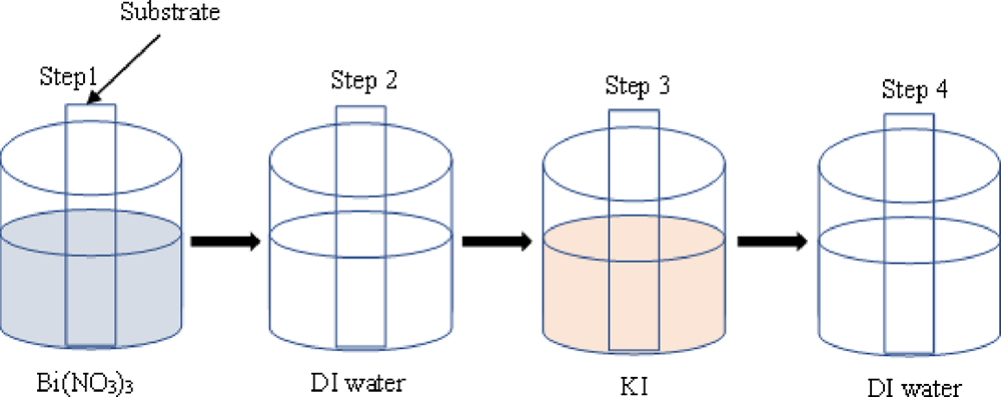
One SILAR cycle for the deposition of BiOI films.

5 mM aqueous solutions of Bi(NO_3_)_3_·5H_2_O and KI were prepared and used as the Bi^3+^ and I^–^ precursors, respectively. The titania films were sensitized with BiOI using SILAR, as follows (Figure 1): (1) the films were immersed in the Bi^3+^ precursor solution for 10 min to adsorb bismuth ions onto the substrates; (2) substrates were rinsed in DI water for 1 min to remove unbounded bismuth ions; (3) the bismuth ion-adsorbed substrates were placed in I^–^ precursor solution for 10 min for reaction between the adsorbed bismuth ions and iodine ions to form BiOI on the surface of the titania films; and (4) the substrates were finally rinsed in DI water to remove unbounded iodine ions. This process completes one SILAR cycle of BiOI deposition. This process was repeated two to eight times to obtain different levels of BiOI on the titania films, after which the films were dried and stored in cleaned containers. The prepared films thickness is between 7 and 8 μm.

### 2.4 Characterization Techniques

The synthesized samples were characterized by the following analytical techniques. The crystalline structures were analyzed with a Bruker D2 phase X-ray diffractometer (XRD) with monochromatized Cu Kα (λ = 1.5406 Å) radiation scanned between 5 and 80° on the 2 theta scale with a scan rate of 0.04°/s. The substrates were set to a rotation speed of 8/min throughout the measurement. The diffuse reflectance of the photocatalysts was measured using a UV–vis spectrophotometer (Shimadzu UV-2600) using a microscope slide as a reference, in the range between 185 and 850 nm. The morphologies of the prepared photocatalysts were analyzed using field emission gun scanning electron microscopy (FEGSEM) and scanning transmission electron microscopy (STEM). The FESEM images of the catalysts were obtained using an FEI Quanta 250 FEGSEM, operated with an accelerating voltage of 15 kV electron beam, while STEM images were collected via JEOL 2100F FEG operated at an accelerating voltage of 200 kV. STEM measurements were performed on FEI Titan Themis operated at 200 kV and equipped with a CEOS DCOR probe corrector, a Super-X energy-dispersive X-ray spectrometer (EDX), and a 4k × 4k Ceta CMOS camera. X-ray photoelectron spectroscopy (XPS) scan was carried out using an Al Kα X-ray source on a Thermo Scientific Theta Probe XPS. Electrochemical impedance spectroscopy (EIS) measurement was carried out in 1 M NaSO_4_ solution using Autolab PGSTAT302N in a three-electrode system without light and at an open-circuit potential. The BiOI and TiO_2_ substrates each coated on FTO (1 cm × 1 cm) served as the working electrode, Ag/AgCl as the reference electrode, and platinum as the counter electrode. The Nyquist plots were measured at frequencies from 0.01 to 100000 Hz.

### 2.5 Photocatalytic Testing

The photocatalytic activities of the synthesized photocatalysts were investigated by the degradation of methyl orange (50 μM), 4-CP (1 mM), and crude oil-contaminated water (200 ppm). 13 W white LED light and a 400 nm UV cutoff filter were used for all the photocatalytic experiments. A quartz cuvette fitted with a lid was used as a reactor to which 0.8 × 3.5 cm films were submerged in methyl orange solution (3 mL) and placed in a light-shielded box. The solution was stirred at 500 rpm on a magnetic stirrer in the dark for 30 min to establish an adsorption–desorption equilibrium (determined as the point at which no further change to the absorbance of the solution occurred) and then illuminated with the LED light with intensity of 340.30 W/m^2^ using a schematic setup shown in Figure S1. The distance between the LED light and the surface of the substrate was 7 cm. The degradation of methyl orange accompanied by its decolorization was determined at every 30 min interval for 180 min by measuring the absorption at 464 nm, scanned through 200–600 nm using a UV–vis spectrophotometer (UNICAM UV 300, Thermal Electron Spectroscopy, Cambridge). The same photocatalytic testing procedure was used for the degradation of 4-CP (3 mL) by measuring the absorption at 280 nm scanned through 200–400 nm.

The 200 ppm crude oil-contaminated water samples were prepared by adding 8 μL of crude oil in 40 mL of DI water. The prepared crude oil–water mixture in a 70 mm ILMABOR glass (reactor) was placed in a light-shielded black box and stirred at 100 rpm on an orbital shaker in the dark for 30 min to disperse the oil in water. 3 cm × 3 cm BiOI/TiO_2_ films were submerged in the crude oil-polluted water in the beaker and stirred in the dark for 30 min and then photocatalyzed for 8, 16, 24, and 48 h with the LED light using a setup, as shown in Figure S2. At the end of each irradiation time, 3 mL of the degraded mixture was pipetted into a quartz cuvette for UV–vis analysis by measuring the absorption at 220 nm, scanned through 190 to 400 nm. The remaining degraded crude oil mixture was then transferred into a separating funnel and extracted with 10 mL of DCM for gas chromatography–mass spectrometry (GC–MS) analysis.

### 2.6 Chemical Oxygen Demand (COD) Analysis

The COD of both undegraded and photocatalytic degraded crude oil-contaminated water samples was determined by using a HACH LCI 400 and measured using a HACH DR 6000 spectrophotometer. The COD HACH tubes containing a mixture of sulfuric acid and potassium dichromate solution were inverted a few times to bring the sediment mixture into suspension. The samples were homogenized by vortex shaking for 60 s at 2500 esc/min to create an emulsion, and 2 mL of aliquot was collected from the middle of the sampling vile and added into the HACH tubes. Reagent blank (deionized water) was also prepared in a similar way. The HACH tubes were then closed and thoroughly mixed by vortexing for a few seconds and then placed in the preheated HACH LT 200 thermodigester and digested at 148 °C for 2 h. At the end of the digestion, the tubes were left for 30 min in the digester after which they were removed and allowed to cool to room temperature for 40 min and the COD was then measured using the spectrophotometer.

### 2.7 GC-MS

An Agilent 7890A GC coupled to an Agilent 7693 autosampler and XL EI/CI MSD with a Triple-Axis detector was used. The column (Agilent HP-5ms) dimension is 30 m × 250 μm × 0.25 μm. The injection volume was 1 μL ran in a splitless mode, and the oven program was as follows: started at 40 °C held for 4 min, ramped at 20 °C/min until a final temperature of 320 °C, and held for 10 min. The carrier gas used was helium gas with a flow rate of 1 mL/min with an ionization temperature between 230 and 250 °C and a quadrupole mass analyzer.

## 3 Results and Discussion

### 3.1 TiO_2_ Film Deposition and BiOI SILAR Sensitization

**Figure 2:**
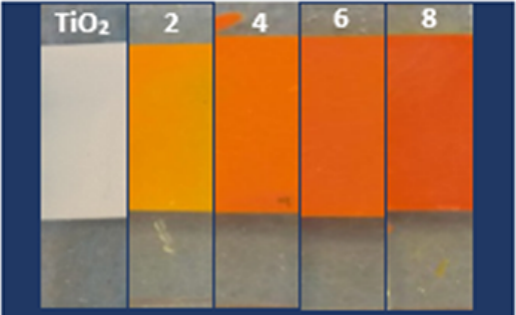
Photographs of the TiO_2_ film and BiOI films deposited using 2, 4, 6 and 8 SILAR cycles.

Commercially available titania paste consisting of a blend of 20 nm active anatase and 450 nm larger anatase particles usually used as a scatter layer in dye-sensitized solar cells was used to produce the mesoporous titania films. This is used to trap the incident light to enhance the light interaction with the photocatalyst. The paste formed white films of titania particles with an interconnected network. The titania films upon SILAR sensitization with BiOI changed from white to orange color, signifying deposition of BiOI on the films' surface. As the number of SILAR cycles increased, the orange color got intense, as shown in Figure 2.

### 3.2 Characterization of Photocatalysts

**Figure 3:**
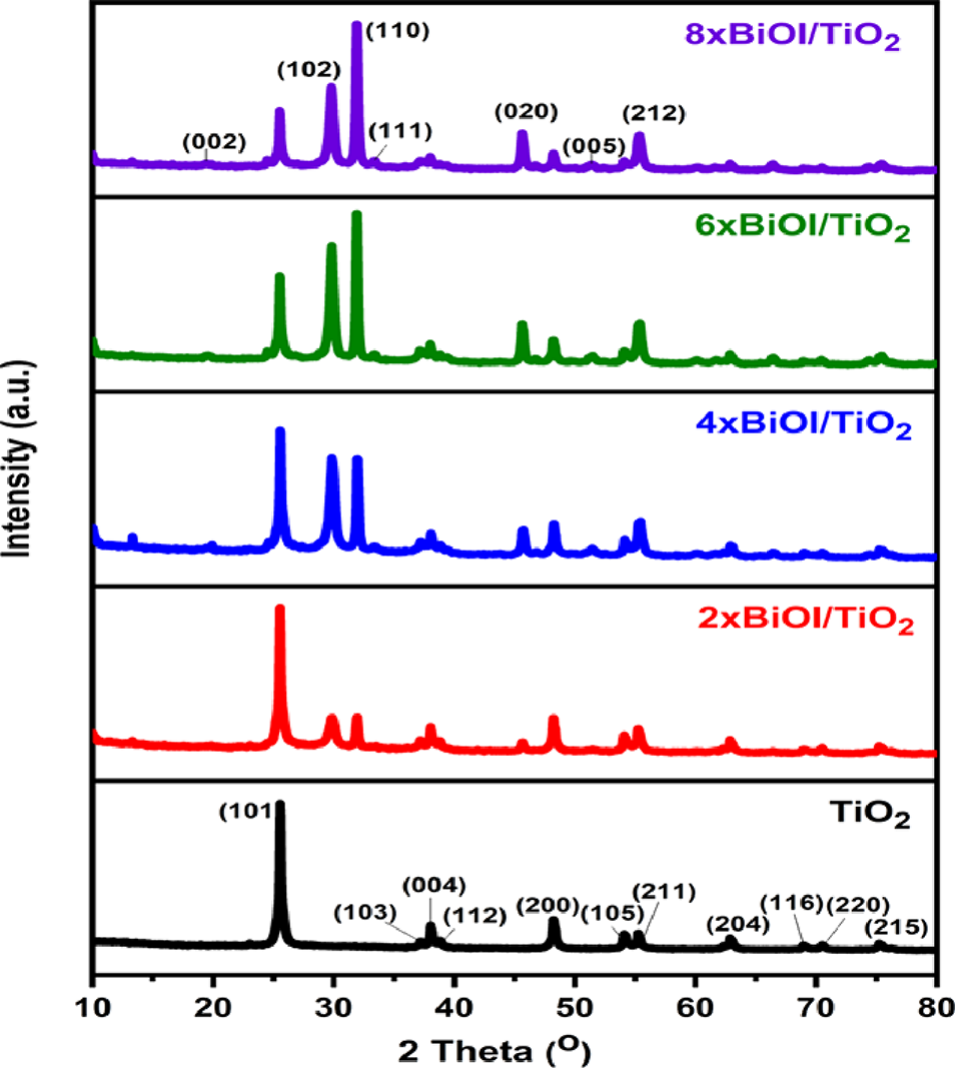
XRD patterns of BiOI/TiO_2_ nanocomposites.

The XRD patterns of the plain TiO_2_ nanoparticle layer and BiOI/TiO_2_ nanocomposites are shown in Figure 3, which refer to the crystallinity and phase composition of the synthesized photocatalysts indexed to the typical tetragonal anatase TiO_2_ (JCPDS 01-084-1286)^44^ and tetragonal BiOI (JCPDS 73-2062; JCPDS 10-0445).^24^ The diffraction peaks belonging to the TiO_2_ phase lie at 2θ: 25.58, 37.4, 38, 38.98, 48.4, 54.18, 55.38, 63, 69, 70.6, and 75.4° corresponding to the (101), (103), (004), (112), (200), (105), (211), (204), (116), (220), and (215) planes,^44,45^ respectively. In the XRD patterns of BiOI/TiO_2_, three prominent peaks at 2θ: 29.82, 32.07, and 45.78° as compared with the XRD pattern of the plain TiO_2_ nanoparticle, which correspond to planes (102), (110), and (020), respectively, were clearly observed. Other lesser-intensity peaks were also observed at 2θ: 19.46, 33.39, and 51.42° corresponding to the (002), (111), and (005) planes, respectively. The peak at 55.36° corresponding to the (212) plane is seen to overlap with the (211) plane of TiO_2_ evident in the peak height. These distinguishable peaks refer to the crystalline BiOI of the tetragonal structure (JCPDS 73-2062; JCPDS 10-0445).^18,24^ This result is consistent with others reported in literature.^18,24,44,45^ Increased peak intensities were observed with an increased number of SILAR cycles indicating thicker BiOI deposition.

**Figure 4:**
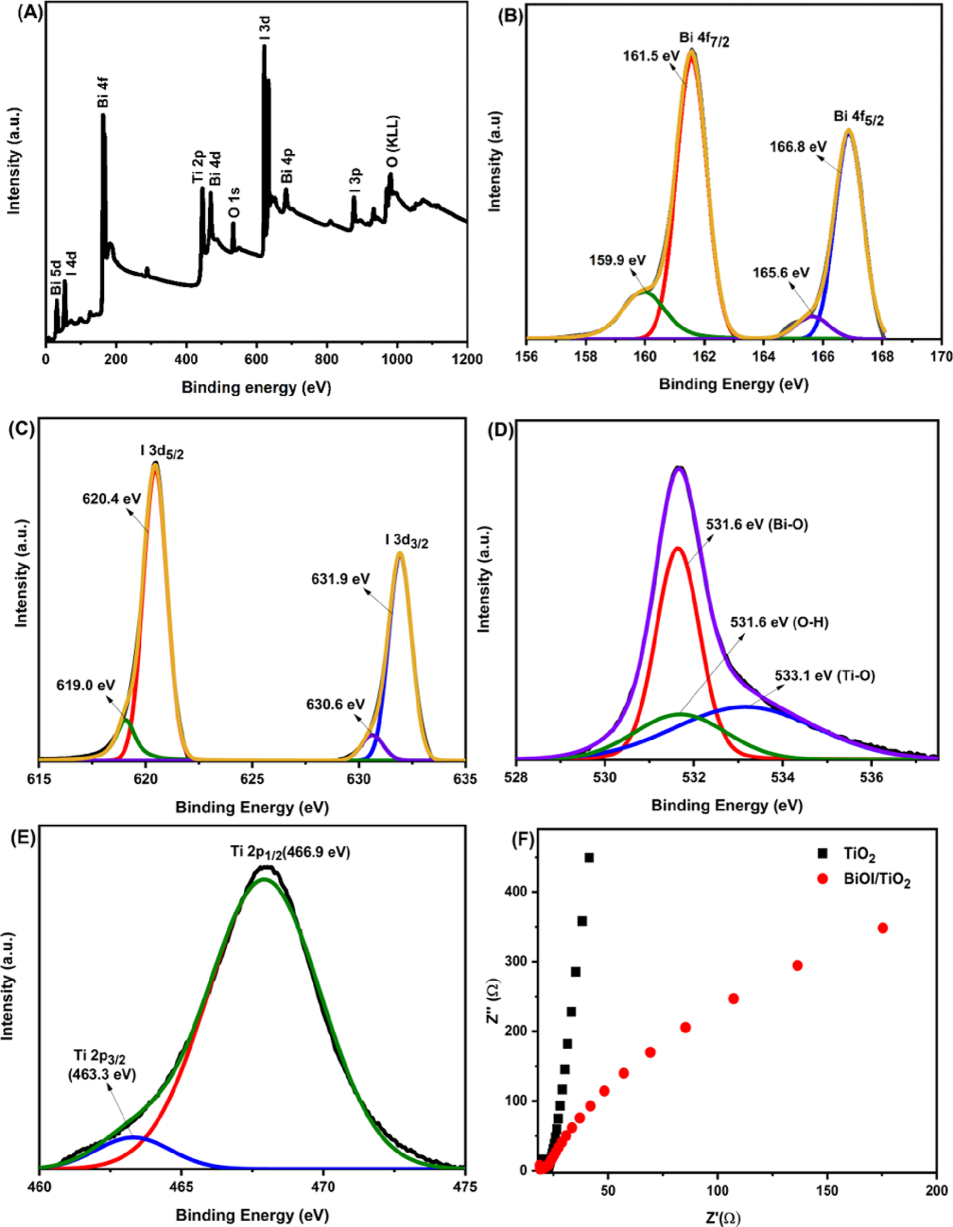
XPS survey of the samples (A) and high-resolution XPS spectra of (B) Bi 4f, (C) I 3d, (D) O 1s (E) Ti 2p, and (F) Nyquist plots of TiO_2_ and 4 × BiOI/TiO_2_ films.

The chemical states and elemental compositions of TiO_2_ and BiOI/TiO_2_ were characterized by XPS, as shown in Figure 4. The Au 4f peak at 84.5 eV (reference) was used to calibrate all the peak positions. The survey spectra of BiOI/TiO_2_ (Figure 4A) show that Bi, I, O, and Ti are present. Compared with TiO_2_, additional peaks of Bi and I were found in BiOI/TiO_2_ along with the Ti and O peaks. All the sample spectra were deconvoluted, and the Voigt fitting method was used to fit the peaks. High-resolution spectra of Bi 4f shown in Figure 4B indicate that Bi 4f was deconvoluted into two doublets (4f_7/2_ and 4f_5/2_) corresponding to 161.5 and 166.8 eV, respectively, and they are characteristic of Bi^3+^ in BiOI.^46−49^ The satellite peaks at 159.9 and 165.6 eV can be ascribed to metallic Bi due to the presence of oxygen vacancies in the system, which is consistent with reported values for BiOI.^28,46−51^ The I band was also deconvoluted into two doublets (Figure 4C) with the distinctive peaks located at 620.4 and 631.9 eV corresponding to I 3d_5/2_ and I 3d_3/2_,^47,52,53^ respectively. Reports have it that bismuth oxide^52^ and TiO_2_^53,54^ doped with I exhibit such doublet deconvolution. The signals of O 1s are at 531.6, 533.1, and 531.6 eV attributed to the Bi–O bonds in [Bi_2_O_2_]^2+^ slabs of BiOI,^28,46,47,51^ Ti–O bonds of TiO_2_,^28,46^ and O–H bonds of the surface-adsorbed water,^46,47,51^ respectively, as seen in Figure 4D. Meanwhile, the two peaks of Ti 2p with binding energies at 463.3 and 466.9 eV correspond to Ti 2p_3/2_ and Ti 2p_1/2_,^55,56^ respectively (Figure 4E). The results therefore confirm successful modification of TiO_2_ nanoparticles with BiOI.

To examine the electronic structure of the prepared photocatalysts, EIS measurements were carried out and the Nyquist plots obtained are shown in Figure 4F. Compared with TiO_2_, BiOI/TiO_2_ shows a smaller radius, which reflects lower charge transfer resistance, indicating higher charge transfer efficiency.^57,58^

**Figure 5:**
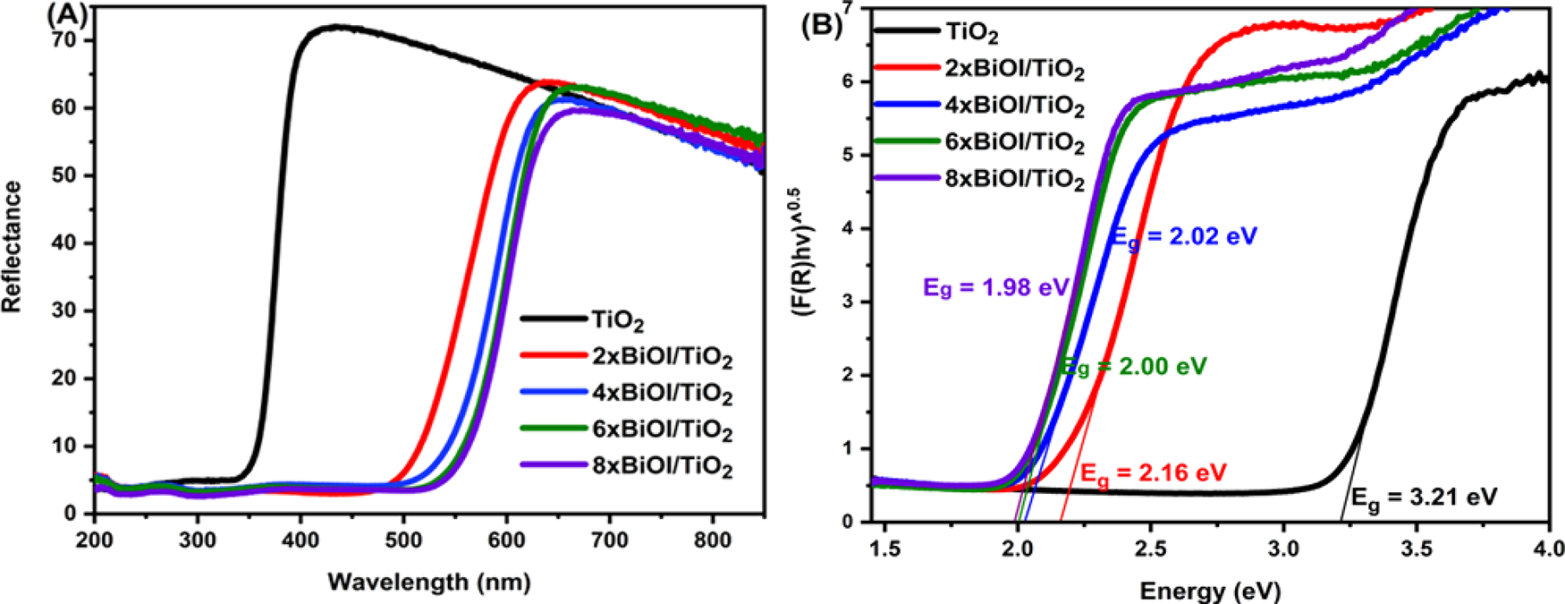
(A) UV–vis diffuse reflectance spectra and (B) Tauc plots of the prepared catalysts.

The diffuse reflectance and optical band gap of the photocatalysts were obtained from UV–vis DRS. From Figure 5A, the diffuse reflectance spectrum of bare TiO_2_ particles absorbs in the UV region with an absorption edge at about 353 nm, which is common for plain TiO_2_. Compared to TiO_2_ nanoparticles, BiOI-sensitized TiO_2_ catalysts exhibit obvious red shifts of the absorption edge with strong absorption of the visible light nearly to the whole visible region, showing smaller band gaps (Figure 5A). The absorption in the visible region was observed to increase with an increase in the number of SILAR cycles. The band-gap energies of the samples were obtained from Tauc plots according to the Kubelka–Munk formula given in eq 1:
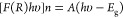
1where *F*(*R*), *h*, υ, *n*, *A*, and *E*_g_ are the absorption coefficient, Planck’s constant, incident light frequency, type of transition (*n* = 1 for direct transition and *n* = 1/2 for indirect transition), a constant, and the band gap, respectively. TiO_2_ and BiOI are known to have indirect transition;^59^ hence, the band gap was determined from the Tauc plot based on [*F*(*R*)*h*υ]^1/2^ versus photon energy (*h*υ) and extrapolating the Tauc plot to the energy axis gives the band gaps for the synthesized nanocomposite catalysts, as shown in Figure 5B. The band gap was observed to decrease with the increase in SILAR cycles.

**Figure 6:**
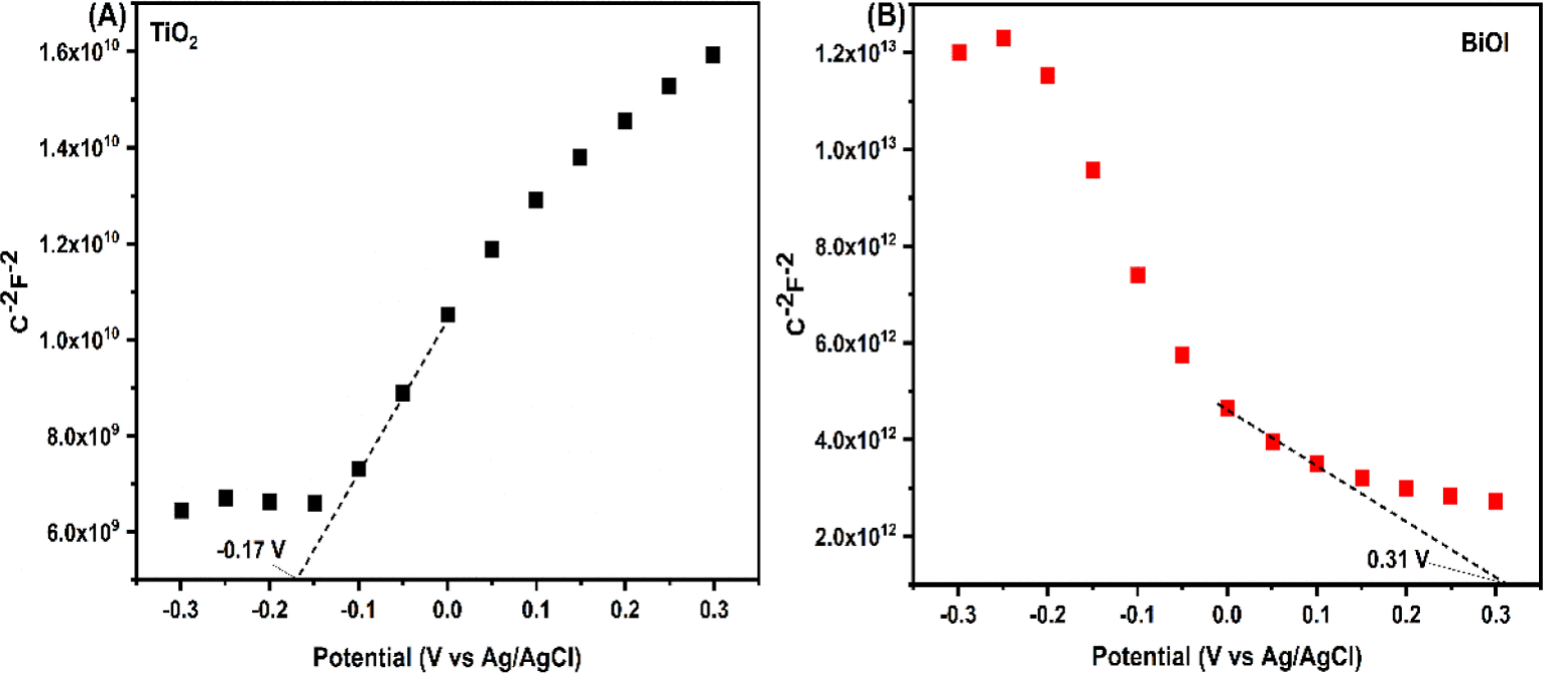
Mott–Schottky plots of (A) TiO_2_ and (B) BiOI on TiO_2_.

Mott–Schottky measurements were performed to determine the semiconductor type and flat-band potential (*E*_fb_) of the prepared TiO_2_ and BiOI, and the plots are displayed in Figure 6. The slopes of the Mott–Schottky plots for TiO_2_ and BiOI are positive and negative, respectively, indicating that the prepared TiO_2_ is an n-type semiconductor whereas BiOI is a p-type semiconductor.^37^ The Fermi levels (*E*_f_) of the photocatalysts were also estimated using the Mott–Schottky plots, and they were found to be −0.17 and 0.31 V for TiO_2_ and BiOI, respectively. This is because the flat-band potential of the photocatalysts in the electrolyte solution is almost the same as the Fermi level of the photocatalysts.^60,61^

**Figure 7:**
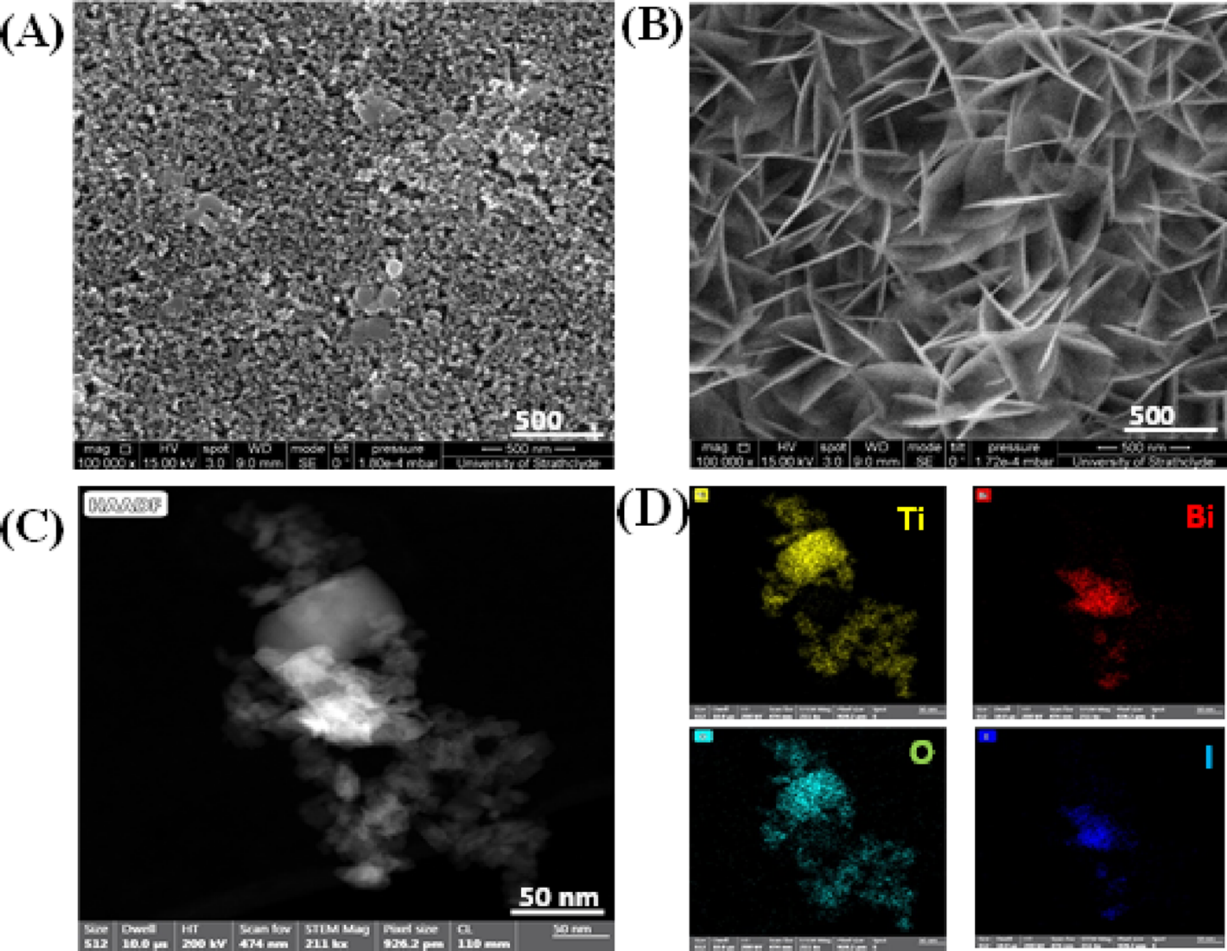
SEM images of (A) TiO_2_, (B) BiOI/TiO_2_, (C) HAADF of BiOI/TiO_2_, and (D) TEM elemental mapping BiOI/TiO_2_.

Figure 7A,B shows the FEGSEM images of the as-prepared TiO_2_ and 4 × BiOI/TiO_2_ photocatalysts. From the FESEM images, typical features of the TiO_2_ films revealed a spherical morphology of the coated nanoparticles. Upon SILAR decoration with BiOI, nanoflakes or plate-like morphologies were seen coated all over the surface of the TiO_2_ films, and as the number of SILAR cycles increases, the plate structures become larger and are more densely packed, as shown in Figure S3. This result agrees with others reported in literature.^18,34,62^

STEM was used to further investigate the microstructure of the 4 × BiOI/TiO_2_ heterojunction. Figure 7C,D gives high-angle annular dark-field (HAADF) images and elemental mapping, which revealed the formation of BiOI/TiO_2_ with good contact between the titania particle and BiOI nanoplates. The elemental maps further confirmed that bismuth and iodine are dispersed on the titania particles by the SILAR method.

**Figure 8:**
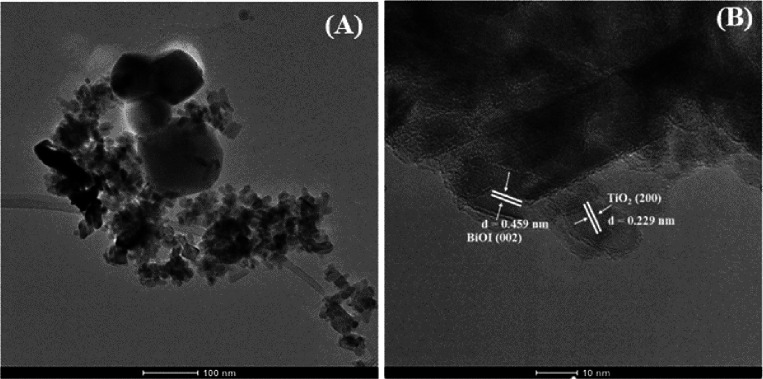
TEM (A) and HRTEM (B) images of BiOI/TiO_2_

To further understand the heterojunction interface, TEM and HRTEM images were obtained, as shown in Figure 8. The BiOI nanoplates (black regions) could be seen attached to TiO_2_ (gray regions) as shown in Figure 8A, and the lattice fringes with spacing of 0.229 and 0.459 nm were observed, as shown in Figure 8B, corresponding to the interplanar spacings of the (200) plane and (002) plane of TiO_2_^63^ and BiOI,^64^ respectively. The HRTEM images strongly confirmed the interfacial interactions between BiOI and TiO_2_ nanocomposites, which facilitate charge separation in the binary composite. Clearly, the TiO_2_ sample has a blend of both smaller (∼20 nm) and larger (>20 nm) anatase nanoparticles.

**Figure 9:**
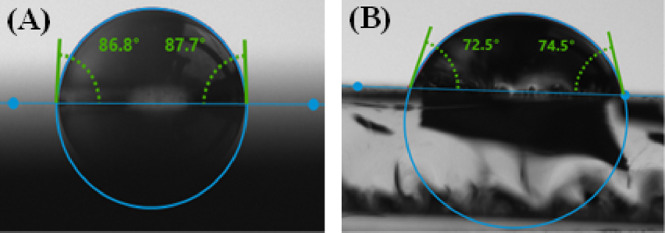
Contact angle of (A) TiO_2_ and (B) BiOI/TiO_2_ films.

To understand the surface hydrophilicity of the as-prepared catalysts for photocatalytic degradation, the water contact angle of the substrates was analyzed. The results from Figure 9 show that both TiO_2_ and BiOI/TiO_2_ display water contact angles less than 90°, indicating that they are hydrophilic^65^ with BiOI/TiO_2_ being more hydrophilic and indicating better wettability. The improvement could be ascribed to the presence of BiOI. The good hydrophilicity of BiOI/TiO_2_ in water treatment is conducive for diffusion of water molecules and with ease of combination to degrade pollutants. Hydrophilic material surfaces with low contact angles (less than 90°) promote the adsorption of pollutants due to their high affinity for polar molecules. This enhanced adsorption increases effective photocatalysis. On the contrary, a hydrophobic material surface will make adsorption of pollutant molecules impossible, which limit photocatalytic reactions.^65,66^

### 3.3 Degradation Evaluation of Methyl Orange and 4-Chlorophenol (4-CP)

The percentage degradation of the prepared photocatalysts against the pollutants is determined according to eq 2:
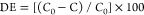
2where DE is the degradation efficiency expressed in percentage, *C* is the concentration at a particular time, and *C*_0_ is the initial concentration.

**Figure 10:**
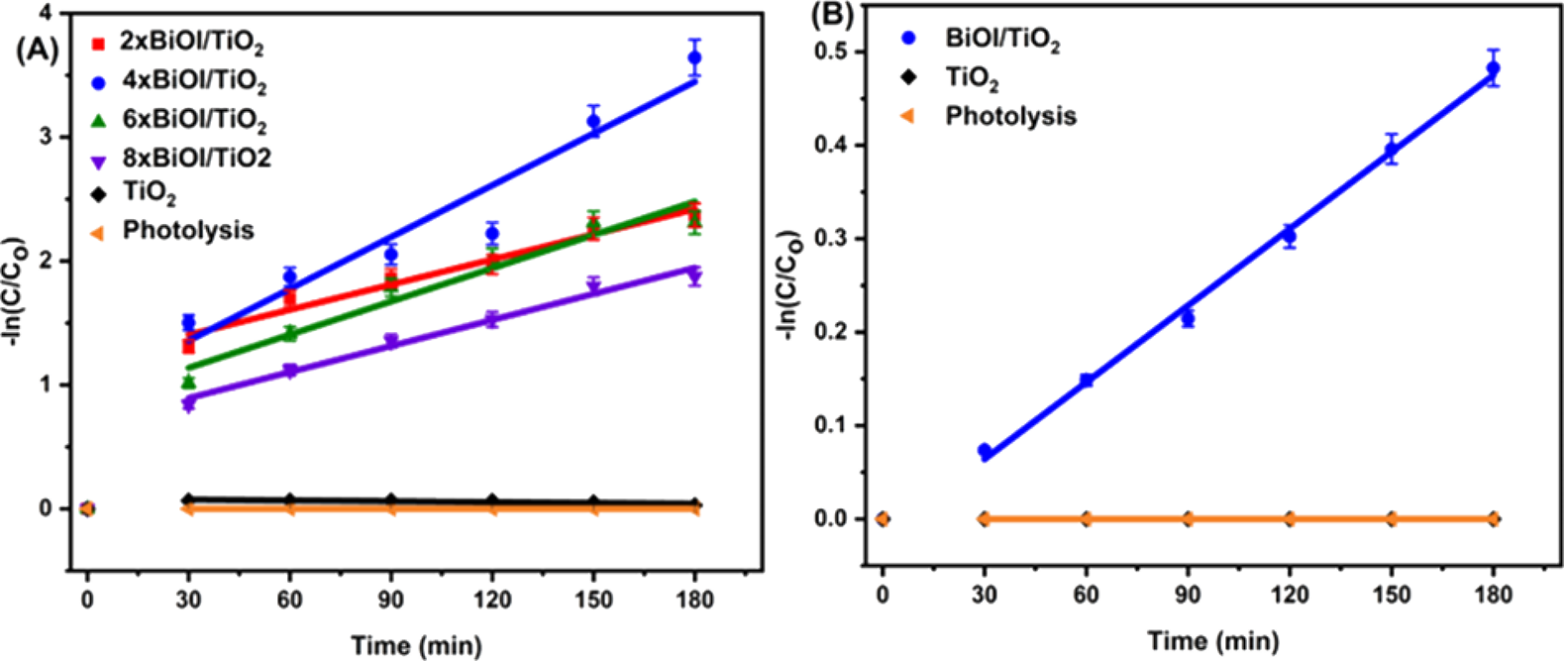
Pseudo-first-order kinetics of TiO_2_ and BiOI/TiO_2_ nanocomposites against (A) methyl orange and (B) 4-CP under visible-light irradiation.

In the degradation of methyl orange, the photocatalytic activity of the composite photocatalysts was found to increase with the number of SILAR cycles up to the fourth cycle, after which a decrease was observed with the rest of the cycles. This may be due to the plates becoming too large for effective charge transfer to the TiO_2_ conduction band. On the other hand, methyl orange was not degraded by photolysis (Figure 10A). Based on the high degradation efficiency (97.38%) of the 4 × BiOI/TiO_2_ substrate against methyl orange, it was chosen as the most efficient for further analysis.

The photocatalytic reactions of the synthesized catalysts were observed to obey pseudo-first-order kinetics according to the Langmuir–Hinshelwood model,^67^ as shown in Figure 10A (expressed in eq 3).
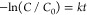
3where *k* is the first-order rate constant (min^–1^) and *t* is time (min). The slope of the plot, −ln(*C*/*C*_0_) versus time, gives the first-order rate constant (*k*). The linear fit for the kinetic rate plots was taken from 30 min of the reaction onward.

To further investigate the photocatalytic activity of 4 × BiOI/TiO_2_, degradation of 4-CP (a colorless UV-absorbing phenolic pollutant) was carried out. This is because the photocatalyst could have specific activity against methyl orange and have a quite different activity toward other pollutants. From Figure 10B, 4-CP was not degraded by pristine TiO_2_ and photolysis while 4 × BiOI/TiO_2_ caused degradation of 4-CP to 38.30% after 3 h of visible irradiation. Comparing the degradation efficiency of 4 × BiOI/TiO_2_ against methyl orange and 4-CP (Table 1), the degradation rate of methyl orange is higher than that of 4-CP and there was no observable degradation of methyl orange by photolysis upon visible-light irradiation, as shown in Figure 10A.

**Table 1:** Photocatalytic Kinetic Values of Methyl Orange and 4-CP by TiO_2_ and BiOI/TiO_2_

	photocatalyst	*K* (10^–3^ min^–1^)	
s. no.		methyl orange	4-CP
1	2 × BiOI/TiO_2_	6.76	
2	4 × BiOI/TiO_2_	13.94	2.68
3	6 × BiOI/TiO_2_	8.94	
4	8 × BiOI/TiO_2_	7.01	
5	TiO_2_	0.067	0.00
6	photolysis	0.00	0.00

In photocatalytic degradation of dye, three possible reaction mechanisms are considered: photolysis, dye photosensitization, or photocatalytic process. In the photolysis process, the excited dye produces photoinduced electrons, which react directly with the oxygen molecule in the reaction system to generate a singlet oxygen atom (^1^O_2_) that operates as an oxidant for the photolysis of dye.^68,69^ In this research work, there was no observable degradation of methyl orange by photolysis upon visible-light irradiation, as shown in Figure 10A. Implying the photolysis mechanism of methyl orange is negligible. In the photosensitization process, the catalyst absorbs the dye and the excited dye produces photoinduced electrons, which migrate to the catalyst conduction band (CB) and react with the oxygen molecule to form a superoxide oxidant.^68−71^ Previous studies reported that dye properties such as dye adsorbability on the catalyst surface, absorbance, and structural stability are responsible for photosensitization of dye.^70,72^ In view of this, the photosensitization of methyl orange (absorbing at λ > 464 nm) was evaluated under visible-light illumination (λ > 400 nm) by using TiO_2_ whose band gap is 3.21 eV (which is responsive at λ = 353 nm). The result shows that little degradation of methyl orange occurs after 180 min of irradiation (Figure 10A). Meaning that photosensitization of methyl orange is negligible. This implies that the degradation of methyl orange in this study is mostly initiated by photocatalytic process. Furthermore, to rule out the possibility of dye photosensitization during photodegradation, BiOI/TiO_2_ film was used for the photocatalytic degradation of 4-CP. The rate order kinetic values from Table 1, reveal that the photocatalytic activity of BiOI/TiO_2_ against 4-CP is lower than that obtained with methyl orange. The low degradation of 4-CP could be a result of the formation of bismuth hydroxide on the surface of the photocatalyst in water^73^ with 4-CP, which has no absorption in the visible region, thereby reducing visible activity, hence less degradation of 4-CP.

### 3.4 Degradation Evaluation of Crude Oil

**Figure 11:**
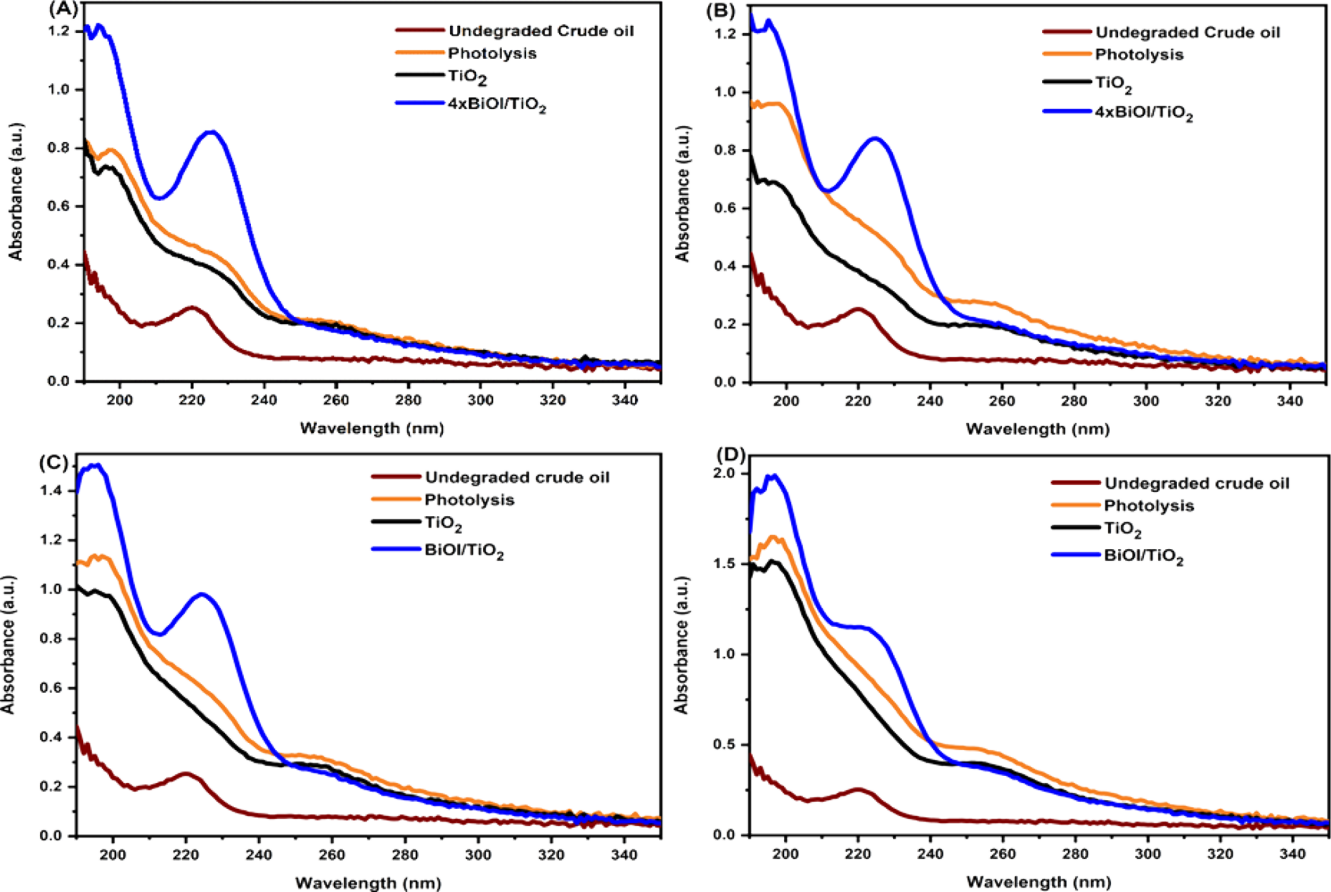
UV–vis spectra for undegraded and photodegraded crude oil-contaminated water in the absence of a photocatalyst (photolysis) and presence of photocatalysts (TiO_2_ and BiOI/TiO_2_) under visible-light irradiation for (A) 8, (B) 16, (C) 24, and (D) 48 h.

UV–vis spectrophotometry was used to determine the concentrations of crude oil before and after photocatalytic degradation. Usually, a decrease in the absorbance peak means a decrease in concentration. However, this was not exactly the case with crude oil, because it is insoluble in water. Figure 11 presents the UV–vis spectra of the crude oil samples, which show that crude oil absorbs at a wavelength range of 206–240 nm with a maximal absorption wavelength at 220 nm. This agrees with the result obtained by Li et al.^9^ The initial dispersion of the crude oil in water was aided by shaking the mixture on an orbital shaker at 100 rpm in the dark for 30 min followed by submerging the catalyst and stirring for another 30 min in the dark. Upon irradiation of the crude oil–water sample, it was found that the absorption peak begins to broaden and increase in intensity with time of irradiation with the appearance of two new peaks around 196 and 253 nm. The increase in the original peak and appearance of the new peaks indicate that the crude oil undergoes more dispersion and degradation under visible-light irradiation,^9^ respectively. Comparing the degradation spectra in the absence of a photocatalyst (photolysis) and in the presence of photocatalysts (TiO_2_ and BiOI/TiO_2_), the absorption maxima of the three peaks continue to increase with time, indicating an increase in the rate of dispersion of the crude oil in water, and it was observed that BiOI/TiO_2_ possessed an outstanding dispersing activity for crude oil than TiO_2_, which is reasonable to presume here that more water-soluble crude oil fractions were dissolved and degraded.

**Figure 12:**
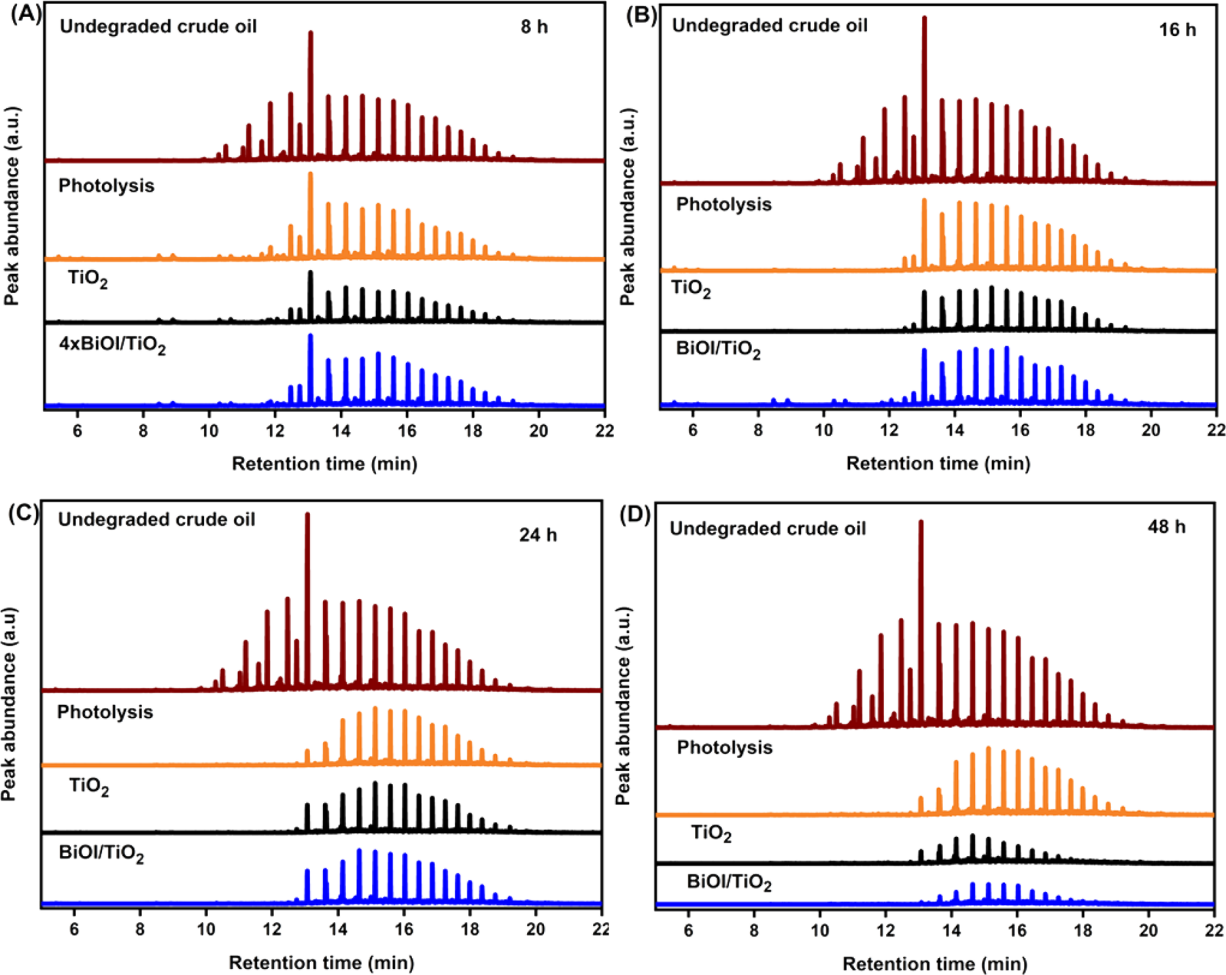
GC-MS ion chromatogram of undegraded and photodegraded crude oil via photolysis and photocatalysis (TiO_2_ and BiOI/TiO_2_) at different times (A) 8, (B) 16, (C) 24, and (D) 48 h.

**Figure 13:**
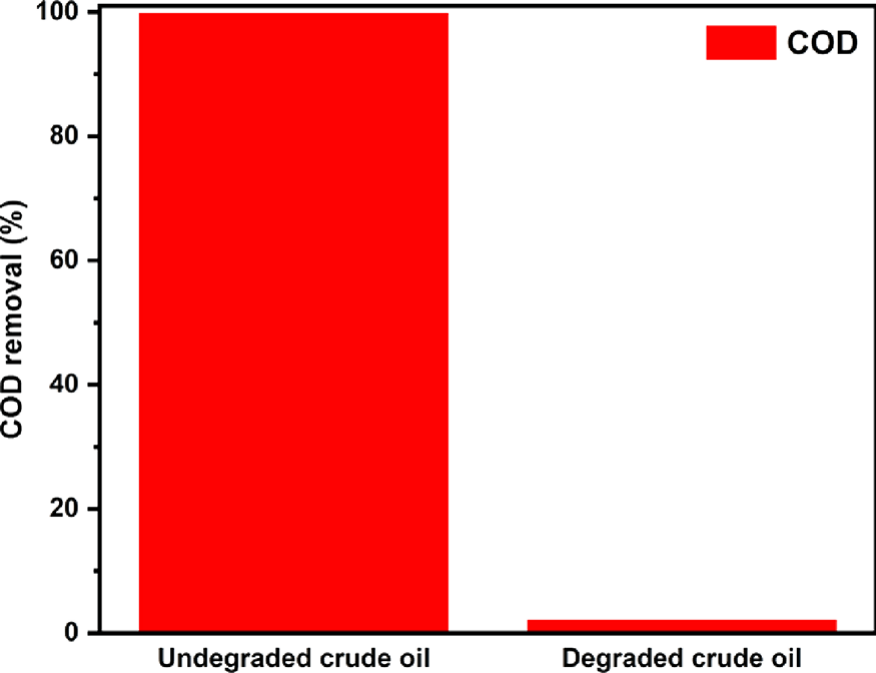
Chemical oxygen demand (COD) value (%) of undegraded and photocatalytically degraded crude oil using BiOI/TiO_2_ under visible-light irradiation.

To further explore the level of changes in composition of the crude oil due to degradation, GC-MS measurements were carried out on undegraded and photodegraded crude oil (using photolysis and photocatalysis), as shown in Figure 12. The GC-MS chromatogram in Figure 12 was selected at mass 57 and 91 to show the chromatographic peaks attributed to the alkane and PAHs being the major compounds of crude oil. *n*-Alkane standards (C_7_–C_40_), polyaromatic hydrocarbon standards, and NIST MS Search 2.0 were used to identify and name the major peaks in the study samples. The degradation percentage of the crude oil components was determined according to eq 2 based on the individual peak area of each sample. The results of the mass chromatograms suggest that the crude oil compositions were in the range of *C*_11_–*C*_29_ (Table S1). Compounds below *C*_20_ were completely decomposed with BiOI/TiO_2_, while the high molecular weight alkanes require a longer time to be mineralized by the chain cleavage step-by-step reaction. Generally, the results show that all the samples exhibited an extensive exponential decrease with time (8, 16, 24, and 48 h) of visible irradiation as seen in their peak intensities with the appearance of new peaks at 5.432, 8.432, and 8.892 min at the eighth hour of degradation, which were identified to be octane, 2-methyl octane, and undecane, respectively, by all the degradation methods. While the new identified peaks disappeared at the 16th hour with TiO_2_, the peaks rather increased in intensity with BiOI/TiO_2_ indicating more degradation, which later disappeared at the 24th hour. Comparing Table S1 and Figure 12, BiOI/TiO_2_ degraded more of the soluble crude oil fraction than TiO_2_ shown by the disappearance of some existing compounds, the diminished concentration of compounds, and the appearance of new peaks indicating severe degradation. It was also observed that photodegradation occurred even without the presence of a photocatalyst (photolysis). This observation is in agreement with the report that after oil spill, some of the crude oil components (mostly the paraffins) are lost through evaporation and photo-oxidation, which is dependent on the light intensity.^3^ However, the presence of a photocatalyst accelerated the photodegradation process and BiOI-sensitized TiO_2_ produced an advantageous synergistic effect on the degradation of the crude oil (85.62%) by enhancing dispersion of the crude oil, thereby making them available for photocatalytic degradation compared to TiO_2_ (70.56%). This superior efficiency of BiOI-modified TiO_2_ could be due to the formation of the hierarchical heterojunction between the two catalysts, which enhances the separation of the charge carriers at the interfaces of the photocatalysts thereby significantly decreasing recombination of the photogenerated charge carriers and promoting photocatalytic activity. Even though TiO_2_ was observed to degrade the crude oil components to a good degree, this was observed to be mostly due to adsorption (Figure 13) of the crude oil fraction onto the catalyst surface and little photolysis.

To further understand the toxicity of the photocatalytically degraded crude oil-contaminated water, COD analysis was carried out to determine the mineralization level of the samples and the results were calculated using eq 2 and are presented in Figure 13. The COD value for the photodegraded sample was observed to be 2.31% of the original 100% undegraded crude oil, signifying 97.69% mineralization. The results demonstrate the effectiveness of the synthesized catalyst for the degradation and mineralization of crude oil in water.

**Figure 14:**
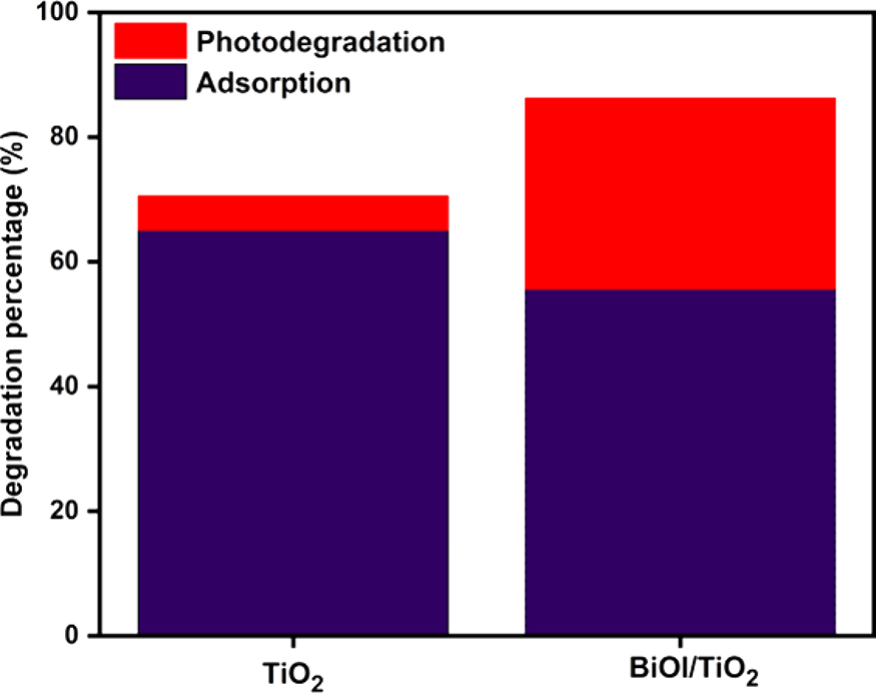
Adsorption coupled with photodegradation of crude oil against TiO_2_ and BiOI/TiO_2_ under visible-light irradiation.

In the adsorption study of the prepared catalysts, TiO_2_ was observed to adsorb crude oil more than BiOI/TiO_2_, as shown in Figure 14. This could be due to the large surface area of TiO_2_ (Figure 7A), which was completely covered by sensitizing with BiOI, which has a smaller surface area (Figure 7B) compared to TiO_2_. This indicates that crude oil degradation by BiOI/TiO_2_ is due to its visible-light activity. Irradiation time also played a significant role in the degradation of the crude oil with the photocatalysts, as seen in Figure 12, indicating that 200 ppm crude oil-polluted water could be completely mineralized within 72 h of photocatalytic degradation using the designed BiOI/TiO_2_.

In the photocatalytic decomposition of crude oil, BiOI/TiO_2_ absorbs visible light, which excites electrons (e^–^) from its valence band to the CB with energy of the photon equals or greater than the band gap of the photocatalyst. Consequently, oxidizing sites called holes (h^+^) are formed in the valence band as well as a reducing site (e^–^) in the CB (eq 4). The photogenerated holes captured on the surface of the photocatalyst undergo charge transfer with surface-bound hydroxyl (OH^–^) species or adsorbed water molecules to form reactive **·**OH radicals shown in eq 5 and eq 6. Even though the degradation mechanisms of the crude oil hydrocarbons were not studied due to the complexity of crude oil components, photocatalytic degradation mechanism, pathways, and intermediates of hydrocarbons (PAHs and alkanes) have been previously studied and reviewed^11,42,43^ and the primary oxidant in the photocatalytic system is the hydroxyl radical, as shown in eqs. 7–12). With the generation of free radicals resulting from the photocatalytic reaction with the hydrocarbons, several reactions such as bond breaking, ring opening, hydroxylation, and ketolysis may occur, which can produce a number of intermediates before final mineralization to carbon dioxide and water.^11,42,43^ However, according to Heller,^74^ the hydroxyl radical is required to initiate the oxidation process by abstracting hydrogen from the organic molecule to form water and organic radical (R**·**CH_2_) while molecular oxygen (O_2_) is the actual oxidizer. The molecular oxygen reacts with the organic radical formed by the reaction of the hydroxyl radical with the organic molecule to generate an organoperoxy radical (RCH_2_OO·), which then reacts with hydroperoxyl radical (**·**OOH) to form organohydrotetraoxide (RCH_2_OOOOH), which then mineralizes to products (eqs. 13–15). The hydroperoxyl radical comes from the reaction of the second molecular oxygen with H^+^ and a photogenerated electron. This is because, for every absorbed photon by the photocatalyst, two molecules of O_2_ are activated.^74^
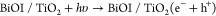
4
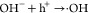
5
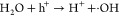
6
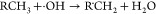
7
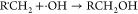
8
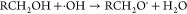
9
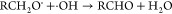
10
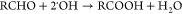
11
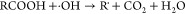
12
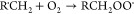
13
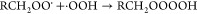
14
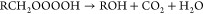
15

### 3.5 Comparison of the Synthesized Photocatalyst with Other Photocatalytic Systems

The photocatalytic activities of the synthesized BiOI-TiO_2_ using the SILAR method in this work was compared with other previous reports on BiOI-TiO_2_ and other TiO_2_-based photocatalysts on crude oil/oily wastewater degradation, and it compares well as shown in Table S2 and S3 even though the target pollutants and conditions differ. The efficiency of the present system was also compared with other non-TiO_2_-based visible-light photocatalyst systems on photocatalytic degradation of crude oil pollutants.^41,42,75,76^ From Table 2, while it can be seen that the percentage degradation per time of all the listed catalysts is higher than the results obtained in this study, it is difficult to make a direct comparison due to the differences in the concentrations of the pollutants, sources of the visible light, and water volume. Furthermore, all the reported studies were based on single pollutant per time, which are free from matrix effects from other pollutants compared with the crude oil in this study, which consists of several components. Also, the volume of the extractant (example, DCM) used has a great effect on the concentration of the degradation products due to a dilution effect. For instance, Yang et al.^76^ used 30 mL of DCM to extract 10 mL of the degradation product as compared with the 10 mL used to extract 10 mL in this study. Moreover, the reported catalysts are in powdered form with diverse dosages, while films of 7–8 μm thickness were used in this study and one of the drawbacks of conventional photocatalysts (powdered) is poor separation from solutions, thereby limiting their uses in water treatment. For ease of separation from the solution mixture and reuse, the prepared photocatalyst was immobilized on FTO glass, which also increases the long-term stability of the photocatalyst.

**Table 2:** Comparing BiOI/TiO_2_ Efficiency with Reported Non-TiO_2_-Based Systems for the Degradation of Crude Oil Pollutants under Visible-Light Irradiation^a^

**photocatalyst**	**form/dosage**	**light source**	**target compound**	**water vol. (mL)**	**percentage degradation/time**	**ref**
Pt-GaN:ZnO	suspension	visible light (300 W Xe lamp, λ = 420 nm with a cutoff filter)	PHE, ANT, ACE, BaA (30 mg)	60 mL	100% degradation of PHE, BaA, ANT, and ACE after 1, 3, 6, and 8 h of irradiation, respectively.	(41)
ZnO/Na_2_S_2_O_8_	suspension (150 mg/L)	visible light (8 W Hg lamp, λ = 300–460 nm)	BaP, BaFLU, BghiP, BkFLU, FLU, InD	150 L	100% degradation of BaP, BghiP, FLU, and InD after 2 h and BaFLU and BkFLU after 4 and 8 h, respectively.	(75)
GO/Ag_3_PO_4_	suspension (1 g)	visible light (300 W Xe lamp, λ = 420 nm with a cutoff filter)	NAP, PHE, PYR (600 μg/L)	1 L	82.1% degradation of NAP in 7 min and 100% of PYR in 30 s	(42)
RCD-CTS	suspension (100 mg)	visible/NIR light (300 W xenon lamp, λ = 420 nm with a cutoff filter)	*n*-tetradecane (5 g/L)	10 mL	51.7%, 4 h	(76)
BiOI/TiO_2_	supported on FTO (thickness 7–8 μm)	visible light (white LED light, λ = 400 nm with a cut off filter)	crude oil	40 mL	85.62%, 48 h	This work

a PHE—phenanthrene, NAP—naphthalene, ANT—anthracene, ACE—acenaphthene, BaA—benzo[*a*]anthracene, PYR—pyrene, BaP—benzo[*a*]pyrene, BaFLU—benzo[*a*]fluoranthene, BghiP—benzo[*ghi*]perylene, FLU—fluorathene, BkFLU—benzo[*k*]fluoranthene, InD—indeno[1,2,3-cd]pyrene, RCD-CTS—reduced g-C_3_N_3_H_*x*_^+^ decatungstate.

The SILAR method of synthesis used in this study is a simple method and consumes less energy coupled with other advantages than previously used methods like hydrothermal and solvothermal yet performed efficiently. For example, since each cycle deposits a specific amount of the material, the SILAR technique provides for perfect control of the film thickness and because it is a low-temperature process, it also prevents the substrate from corrosion and oxidation.^77^ Compared with the other methods, the SILAR method offers a straightforward and economical approach to synthesize photocatalytic materials with predetermined properties, making it suitable for a range of photocatalysis applications. Also, the method is capable of large-area fabrication using less time and energy along with having good reproducibility.^77−79^

Based on the comparisons made above, it is evident that the synthesized BiOI/TiO_2_ is an efficient photocatalyst for the degradation of methyl orange, 4-CP, and crude oil hydrocarbons in water.

### 3.6 Determination of Reactive Species

**Figure 15:**
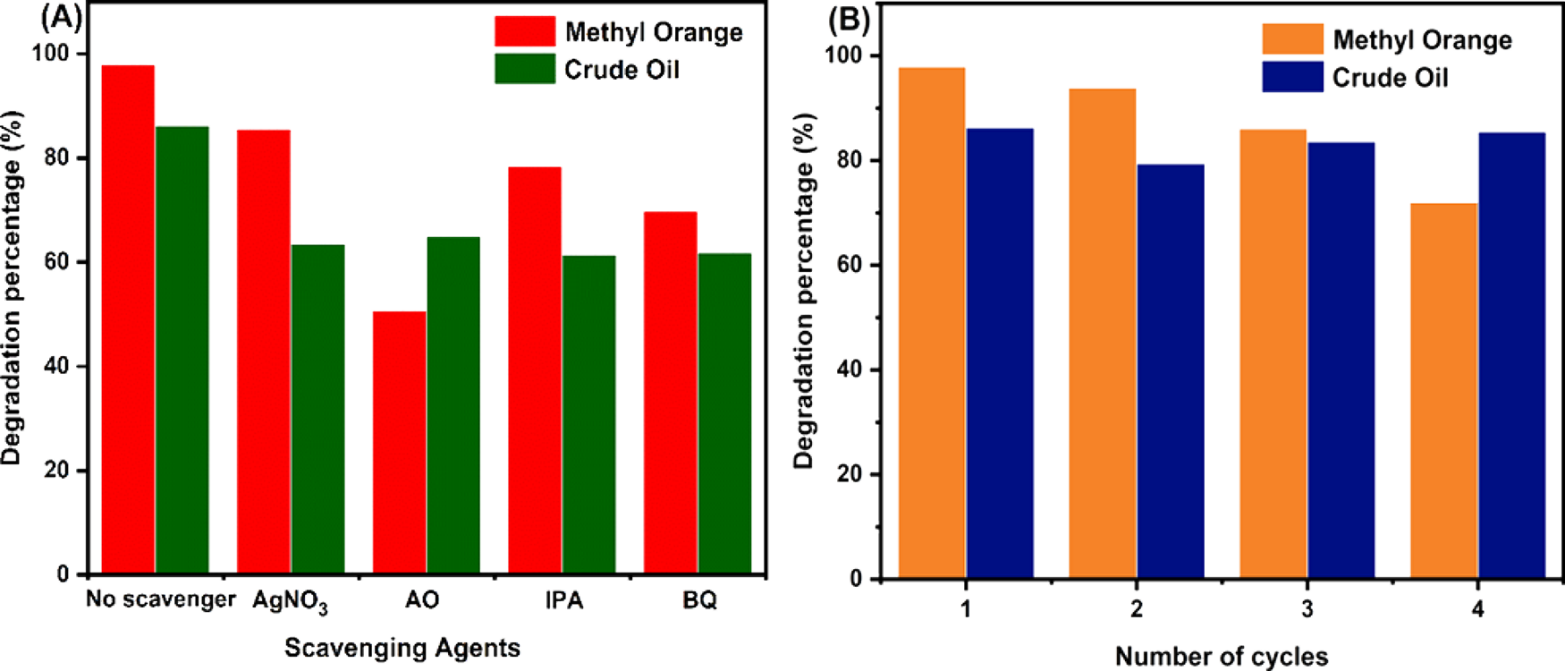
(A) Reactive species tests (AgNO_3_—silver nitrate, AO—ammonium oxalate, IPA—isopropyl alcohol, BQ—benzoquinone). (B) Recycling tests of BiOI/TiO_2_ against methyl orange and crude oil.

It is established that photocatalytic degradation of pollutants depends on the generated reactive species on the photocatalysts surface. The reactive species of BiOI/TiO_2_ was determined to understand its mechanism in photocatalytic degradation of methyl orange and crude oil. This was done by using 10% of 5 mM solution of the scavenging agents [ammonium oxalate (AO), benzoquinone (BQ), IPA, and silver nitrate (SN)] used to scavenge hole (h^+^), superoxide radical (^**·**^O_2_^–^), hydroxyl radical (^**·**^OH), and electron (e^–^), respectively. Typically, 10% of the scavenging agent solution was added to the appropriate volume of methyl orange and crude oil–water samples and photodegraded for 3 and 48 h, respectively. The result from Figure 15A shows that all of the reactive species are active in the photodegradation of the pollutants by BiOI/TiO_2_ under visible-light irradiation. However, h^+^ and ^**·**^O_2_^–^ play more vital roles in methyl orange degradation, as also reported by Cao et al.^68^ while ^**·**^OH and ^**·**^O_2_^–^ play more vital roles in crude oil degradation. This observation agrees with the above degradation mechanism (eqs. 7–15) and the proposed band structure mechanism. To further understand which of the reactive species is important for the photocatalytic degradation, continuous-wave–electron paramagnetic resonance (cw-EPR) measurements were carried out using the most popular spin-trap 5,5-dimethyl-1-pyrroline *N*-oxide (DMPO). Figure S5 shows cw-EPR spectra of the BiOI+TiO_2_+DMPO, TiO_2_+DMPO, fresh DMPO, and DMPO left on bench for 16 h and Figure S6 shows cw-EPR spectra of the BiOI+TiO_2_+DMPO when exposed to a 150 W xenon lamp with a 400 nm filter for different times. Figure S5 reveals that the DMPO-adduct signal, especially the DMPO-R signal, is decreased in intensity significantly when BiOX+TiO_2_ (black and red traces) is mixed with the aqueous solution of DMPO (blue trace). It enhances the visibility and/or increases the formation of the DMPO–OH signal. It is also observed that DMPO-R signals are completely lost (magenta trace) when the freshly prepared DMPO solution was sitting on the bench for ∼16 h—the observed signals are predominantly due to the DMPO–OH adduct whereas Figure S6 reveals that no new DMPO-adduct signals are formed when DMPO solution of BiOI+TiO_2_ (red traces) was exposed to a 150 W xenon lamp with filter. Therefore, it was not possible with the EPR measurements to conclude which of the reactive species is more important for photocatalytic degradation.

### 3.7 Recycling Test

**Figure 16:**
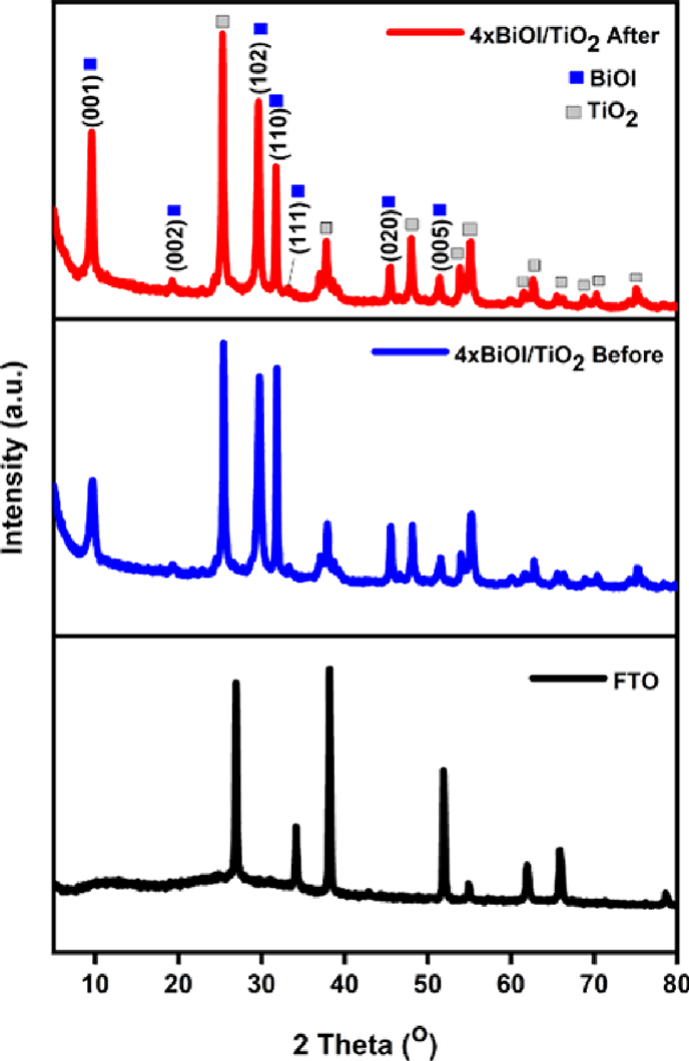
XRD patterns of the BiOI/TiO_2_ nanocomposite before and after photocatalytic reaction.

To check the reusability of synthesized BiOI/TiO_2_, we measured its performance over time. The photocatalyst was reused four times for degrading methyl orange, with each cycle lasting for 3 h, and four times for degrading crude oil, with each cycle lasting for 48 h (Figure 15B). After each cycle, the catalyst was washed with DI water five times and then oven-dried at 100 °C for 60 min. At the end of the fourth cycle, the degradation percentage of methyl orange decreased to 72% from 97.38% indicating 25.94% loss of activity, which could result from clogging of the catalyst surface. However, Cai et al.^37^ reported negligible loss of activity of BiOI/TiO_2_ on cyclic degradation of methyl orange in five cycles for 100 min per cycle whereas Odling and Robertson^18^ in their stability experiment of BiOI/TiO_2_ against 4-CP reported that a bit of instability with the photocatalyst was observed, which is believed to be due to loss of iodine and formation of bismuth hydroxide on the layer surface,^73^ which has no absorption in the visible region, thereby reducing visible activity. This could be the reason for the low photocatalytic degradation of 4-CP compared to the degradation of methyl orange and crude oil in this study. With crude oil, the result shows that after the first cycle of degradation with 85.62%, the catalyst lost 6.22% activity in the second run (79.40%). However, the activity loss was regained in the third run (83.59%) and fourth run (85.49%) with the fourth run almost at the same level of efficiency as the first run. This shows that the catalyst is more stable with crude oil than with methyl orange, which could be because of the oven drying after each cycle causing evaporation of undegraded crude oil

adsorbed onto the catalyst surface as compared to adsorbed methyl orange components, which are not volatile as crude oil components. This could be seen in the color change (Figure S4) of the catalyst after recycling, as the catalyst used for crude oil remains almost the same as the unused compared with the one used for methyl orange degradation. In addition, XRD of both fresh and used (after using for 48 h of visible photocatalytic remediation of crude oil-contaminated water) BiOI/TiO_2_ nanocomposite samples was also carried out to further probe the stability of the photocatalyst. As shown in Figure 16, the structure and phase of the used BiOI/TiO_2_ nanocomposite remained unchanged after the photocatalytic reaction. The excellent stability of BiOI/TiO_2_ may be due to the heterojunction formed between BiOI and TiO_2_.

### 3.8 Band Structure of BiOI/TiO_2_ and Mechanism of Photocatalytic Degradation of the Studied Pollutants

The band structure diagram of the synthesized BiOI/TiO_2_ photocatalyst was constructed by using the atom’s Mulliken electronegativity eqs. 16 and 17) to calculate the band positions of TiO_2_ and BiOI:^26−28,35−37^
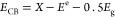
16
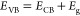
17where *E*_CB_ is the conduction band potential, *E*_VB_ is the valence band potential, *X* is the electronegativity of the semiconductor, which represent the geometric mean of the electronegativity of the constituent atoms (TiO_2_ = 5.81 eV and BiOI = 5.99 eV),^80^*E*^e^ is the energy of the free electrons on the hydrogen scale, which is about 4.5 eV, and *E*_g_ is the band gap determined from diffuse reflectance spectroscopy measurement. From the above information, the *E*_CB_ of TiO_2_ and BiOI were calculated to be −0.3 and 0.48 eV, respectively, while the *E*_VB_ of TiO_2_ and BiOI were calculated to be 2.92 and 2.5 eV, respectively.

**Figure 17:**
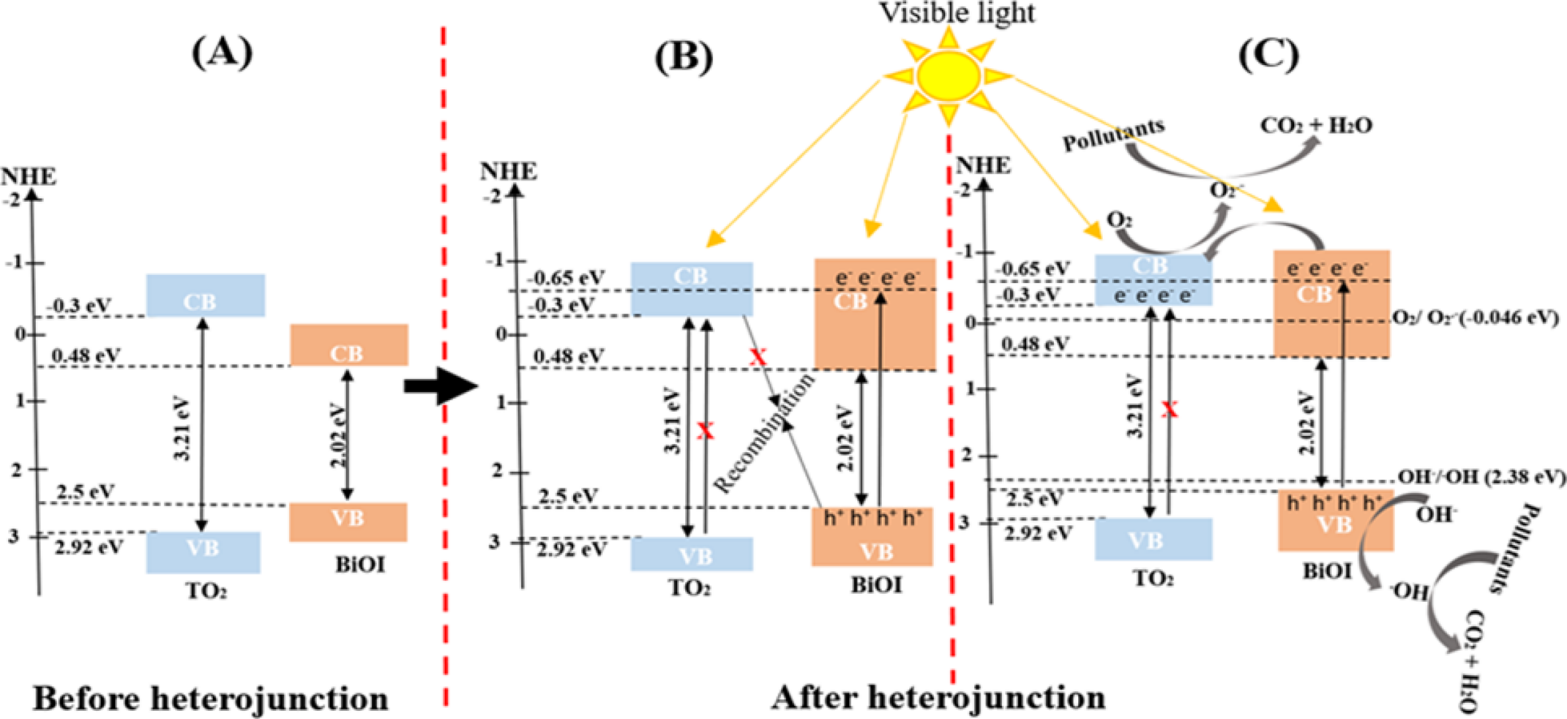
Proposed mechanism of action of the prepared BiOI/TiO_2_ heterojunction.

Photocatalyst band structure is responsible for effective generation and separation of e^–^/h^+^ pairs.^37^ However, from the calculated band structure potential of TiO_2_ and BiOI, it appears that the separation of the e^–^/h^+^ pairs is not favorable in the BiOI/TiO_2_ composite, as shown in Figure 17A, because the CB of BiOI (0.48 eV) lies below that of TiO_2_ (−0.30 eV) and its VB (2.50 eV) lies above that of TiO_2_ (2.92 eV), thereby preventing separation of the photogenerated charges resulting in a high recombination rate of the e^–^/h^+^ pairs. This observation has been reported by many in literature. However, it is important to note that the values calculated are for TiO_2_ and BiOI before the formation of the heterojunction. Upon formation of the junction and Femi-level alignment,^61,81−83^ VB electrons in the BiOI under visible-light irradiation could be excited to a higher potential edge of −0.65 eV (λ > 420) with energy less than 2.95 eV.^24,68^ Consequent with the reformed CB potential edge of BiOI (−0.65 eV), which is the newly formed CB of BiOI resulting from absorption of higher photon energy, it becomes more negative than that of TiO_2_, thereby allowing easy transfer of the photogenerated electrons from the reformed CB of BiOI by means of the internal electric field to that of TiO_2_. Since the CB electrons in the TiO_2_ are more negative than the standard redox potential of O_2_/·O_2_^–^ (−0.046 eV),^37^ it indicates that the electrons on the surface of TiO_2_ can reduce the adsorbed O_2_ on the BiOI/TiO_2_ surface to superoxide (·O_2_^–^), which then degrade the pollutants, while the holes on the VB of BiOI being more positive than the standard redox potential of ·OH/OH^–^ (2.38 eV)^37^ cause oxidation of the OH^–^ into ·OH, which also degrade the studied pollutants. Therefore, it is reasonable to say that ·O_2_^–^ and ·OH are the main reactive species responsible for the photocatalytic degradation of the crude oil pollutant in the BiOI/TiO_2_ heterojunction. Here, TiO_2_ could not be excited by the visible-light illumination and as such a direct Z-scheme heterojunction is not possible (Figure 17B). A direct Z-scheme heterojunction would have been possible if UV light was to be used. With that, upon excitation of the catalysts by UV light and separation of the photogenerated charges, electrons in the CB of TiO_2_ will recombine with the holes in the VB of BiOI, leaving the electrons in the CB of BiOI with strong reduction potential to reduce O_2_ to O_2_^.^ while the holes in the VB of TiO_2_ with strong oxidation potential to oxide OH^–^ to ·OH,^58,83,84^ as shown in Figure 17B. Thus, in these heterojunction composites, TiO_2_ acts as an electron relay semiconductor by accepting electrons from BiOI^58,85^ (Figure 17C), thereby preventing charge recombination. This band arrangement agrees with previously proposed alignment between the TiO_2_ and BiOI heterojunction.^26,27,36,37,86^

## 4 Conclusions

In summary, BiOI/TiO_2_ heterojunction photocatalysts with different deposition levels of BiOI were successfully synthesized via the SILAR method with high visible-light reactive activity than TiO_2_ against crude oil degradation. The formation of BiOI/TiO_2_ was confirmed by XRD, XPS, FEGSEM, TEM, and diffuse reflectance spectroscopy (DRS) analyses. As evidenced by the DRS spectra, the wide band gap of TiO_2_ was successfully sensitized by the narrow band gap of BiOI. The photocatalytic activity of the synthesized photocatalysts with varied levels of BiOI deposition was assessed by the photodegradation of methyl orange, 4-CP, and crude oil-contaminated water under visible-light illumination. Of the various photocatalysts studied, the BiOI/TiO_2_ heterojunction (with 4 SILAR BiOI deposition on TiO_2_) exhibited the best degradation activity as confirmed by its reaction rate order constant, which is 14 and three times higher than that of TiO_2_ for methyl orange and 4-CP, respectively. In the degradation of crude oil, the synthesized BiOI/TiO_2_ showed a higher photodegradation efficiency under visible light than that of TiO_2_, observed to be due to the red shift in the band gap of the BiOI/TiO_2_ heterojunction photocatalyst due to the presence of BiOI. However, photodegradation of crude oil by TiO_2_ was mainly due to adsorption and little photolysis. Detailed scavenging tests confirm that h^+^ and ·O_2_^–^ play more vital roles in methyl orange degradation while ·O_2_^–^ and ·OH play more vital roles in crude oil degradation. The results thus show the potential application of BiOI/TiO_2_ photocatalysis in remediation of crude oil-contaminated water.

## Supporting Information Available

Schematic diagram of the setup for photocatalytic degradation of methyl orange, 4-CP, and crude oil-contaminated water; SEM images of FTO, TiO_2_-coated FTO, and different SILAR cycles of BiOI gown on TiO_2_; images of 4 × BiOI/TiO_2_ samples before and after visible-light photodegradation; cw-EPR spectra of the BiOI+TiO_2_, TiO_2_, fresh DMPO, and DMPO left on a bench for 16 h; cw-EPR spectra of the BiOI+TiO_2_+DMPO was exposed to a 150 W xenon lamp with a 400 nm filter for different times; GC-MS results obtained for the photodegradation processes of crude oil under study; comparison of TiO_2_-based photocatalysts for crude oil/oily water remediation; and summary of previous reports on BiOI/TiO_2_ photocatalysts (PDF)
