# Supplementary material for: Visible-Light-Active BiOI/TiO2 Heterojunction Photocatalysts for Remediation of Crude Oil-Contaminated Water
Source: ACS Omega. 2023 Nov 7;8(46):43556–72. doi: 10.1021/acsomega.3c04359 (PMC10666155; doi:10.1021/acsomega.3c04359)
Supplement: Supplementary file 1 — ao3c04359_si_001.pdf [file ao3c04359_si_001.pdf]

Visible Light Active BiOI/TiO$_2$ Heterojunction Photocatalyst for Remediation of Crude Oil Contaminated Water

Supplementary Information

Blessing Ogoh-Orch, Patricia Keating and Aruna Ivaturi*

Smart Materials Research and Device Technology (SMaRDT) Group, Department of Pure and Applied Chemistry, University of Strathclyde, Thomas Graham Building, 295 Cathedral Street, Glasgow G1 1XL.

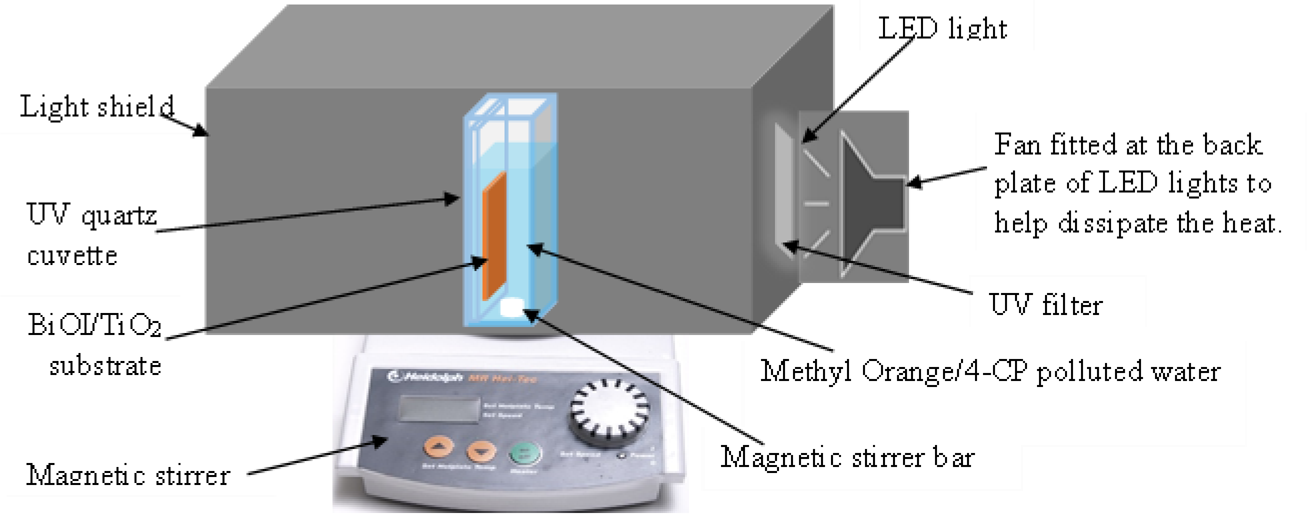

Figure S1. Schematic diagram of the setup for photocatalytic degradation of methyl orange and 4-CP contaminated water

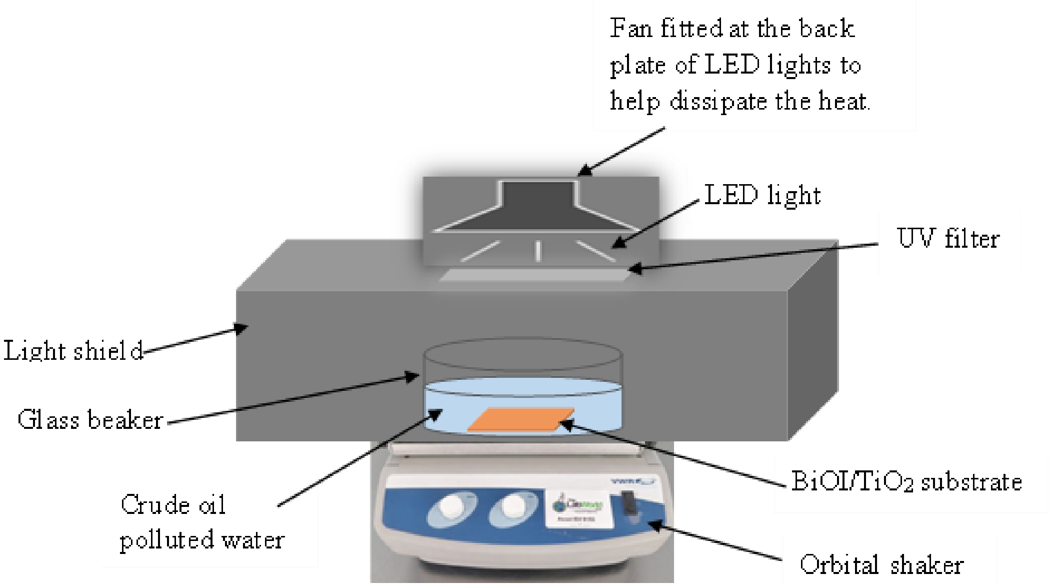

Figure S2. Schematic diagram of the setup for photocatalytic degradation of crude oil contaminated water

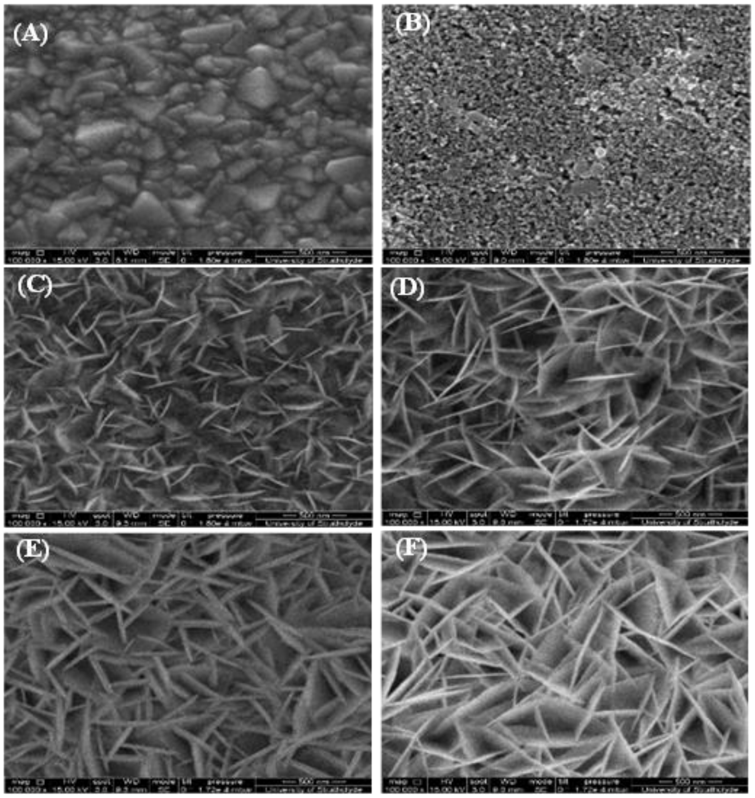

Figure S3. SEM images of (A) FTO, (B) TiO_2 coated FTO, (C) 2xBiOI/TiO_2, (D) 4xBiOI/TiO_2, (E) 6xBiOI/TiO_2 and (F) 8xBiOI/TiO_2

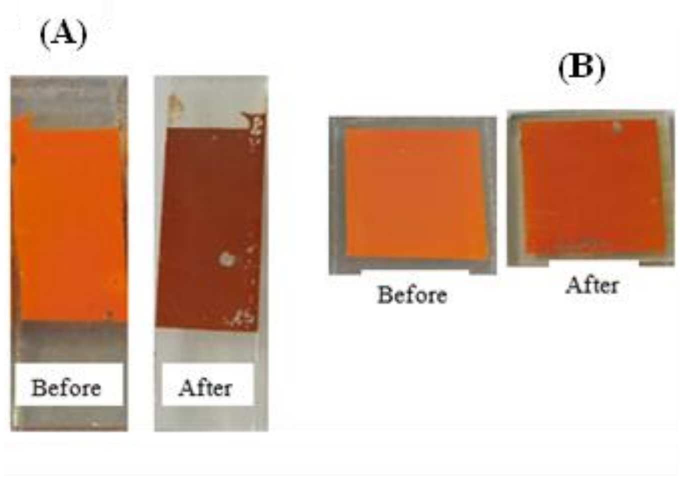

Figure S4. Images of 4xBiOI/TiO_2 samples before and after visible light photodegradation with (A) methyl orange (3 hours) and (B) crude oil (48 hours)

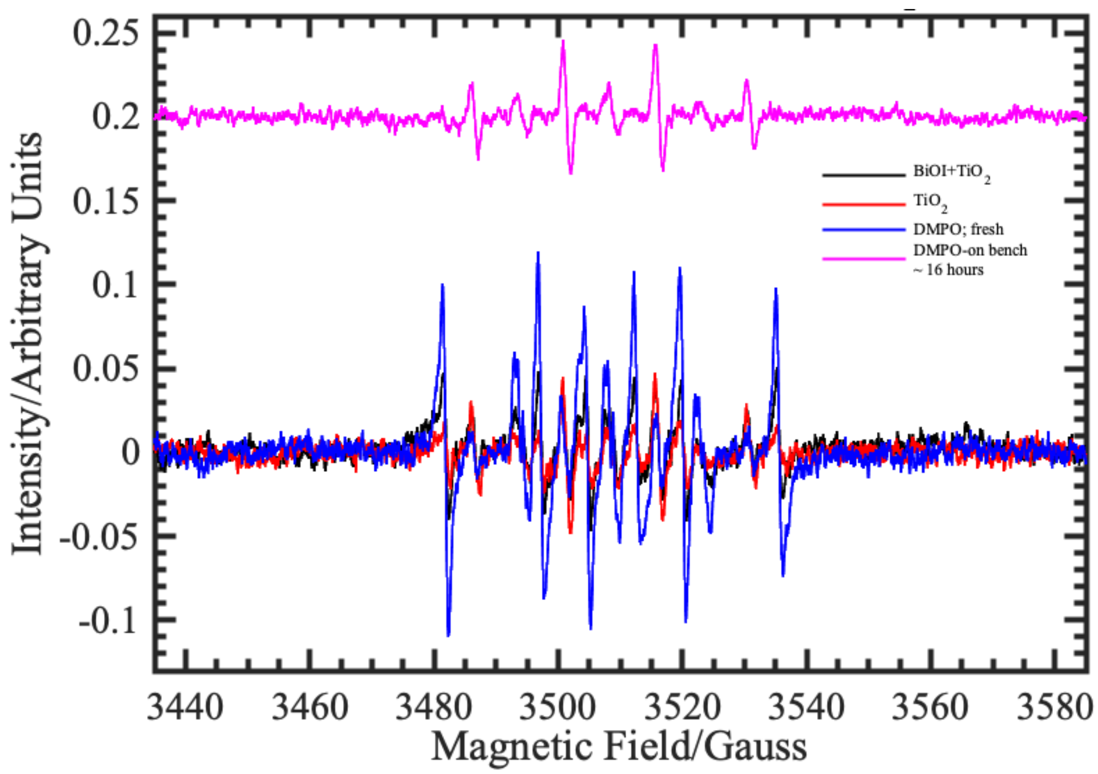

Figure S5. cw-EPR Spectra of the BiOI+TiO_2+DMPO, TiO_2+DMPO, fresh DMPO and DMPO left on bench for 16 hours.

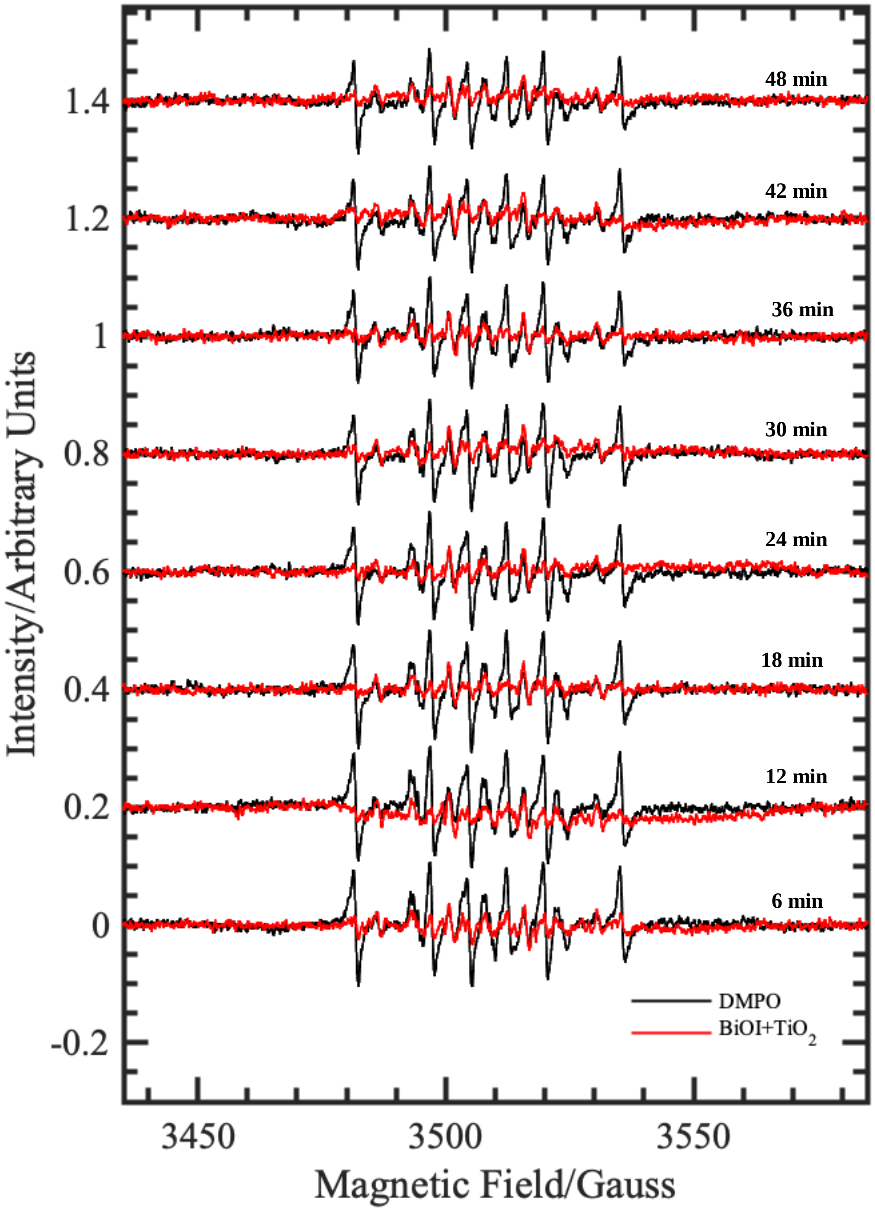

Figure S6. cw-EPR Spectra of the BiOI+TiO_2+DMPO was exposed to 150 W xenon lamp with 400nm filter for different times.

Table S1. GC-MS results obtained for the photodegradation processes of crude oil under study.

Retention Time (min)	Carbon number	Degradation method			Identified compound
		Photolysis	Photocatalysis		
			TiO_2 + visible light	BiOI/TiO_2 + visible light	
10.504	13	100 % degradation	100 % degradation	100 % degradation	Tridecane
11.030	11	,,	,,	,,	1-Methylnaphthalene
11.202	14	,,	,,	,,	Tetradecane
11.595	12	,,	,,	,,	Cyclobutadibenzene
11.857	15	,,	,,	,,	Pentadecane
12.254	12	,,	,,	,,	4-dodecene-6,8,10-triyn-3-one
12.474	16	,,	,,	,,	Hexadecane
12.746	13	,,	,,	,,	Dodecane, 2-methyl
13.0750	17	92 % degradation from 19.23 % to 1.52 %	94 % degradation from 19.23 % to 1.47%	,,	Heptadecane
13.616	18	77 % degradation from 5.98 % to 1.39%	88 % degradation from 5.98 % to 1.10%	,,	octadecane
13.649	20	68 % degradation from 4.06 % to 1.31 %	69 % degradation from 4.06 % to 1.26 %	87 % degradation from 4.06 % to 0.51 %	Hexadecane, 2,6,10,14-tetramethyl
14.083	19	205 % increase from 0.98 % to 2.98	100 % degradation from 0.98 % to 0 %	100 % degradation	Nonadecane
14.144	19	37 % degradation from 5.74 % to 3.61 %	76 % degradation from 5.74 % to 1.38 %	85 % degradation from 5.74 % to 0.85 %	Nonadecane
14.648	20	33 % degradation from 5.47 % to 3.65 %	71 % degradation from 5.47 % to 1.56 %	76 % degradation from 5.47 % to 1.30 %	Eicosane
15.129	21	37 % degradation from 5.21% to 3.26 %	74 % degradation from 5.21 % to 1.37 %	76 % degradation from 5.21 % to 1.23 %	Heneicosane
15.59	22	33 % degradation from 5.19 % to 3.46 %	77 % degradation from 5.19 % to 1.18 %	79 % degradation from 5.19 % to 1.07 %	Docosane
16.032	23	29 % degradation from 4.98 % to 3.53 %	79 % degradation from 4.98 % to 1.06 %	80 % degradation from4.98 4% to 1.0 %	Tricosane
16.455	24	28 % degradation from 4.28 % to 3.09 %	78 % degradation from 4.28 % to 0.92 %	80 % degradation from4.28 % to 0.84 %	Tetracopsane
16.864	25	33 % degradation from 4.62 % to 3.10 %	82 % degradation from 4.62 % to 0.80 %	82 % degradation from 4.62 % to 0.80 %	Pentacosane
17.255	26	38 % degradation from 3.74 % To 2.33 %	100 % degradation	100 % degradation	Hexacosane

17.634	27	19 % degradation from 2.89 % to 2.33 %	100 % degradation	100 % degradation	Heptacosane
17.998	28	0 % degradation from 2.53 % to 2.53 %	100 % degradation	100 % degradation	Octacosane
18.371	29	37 % degradation 1.28 % to 0.81 %	100 % degradation	100 % degradation	Nonacosane

Table S2. Comparison of TiO_2 based photocatalysts for crude oil/oily water remediation.

Photocatalyst	Form	Light Source	Target compound	Pollution source	Photodegradation Activity	Reference
Degussa P25	Suspension	UV (Hg lamp, λ = 254 nm)	Oil emulsion (diesel oil)	Synthetic (distilled & sea water)	92 % and 43 % degradation achieved for synthetic & Sea water respectively	[10]
TiO_2	Suspension	1500 W Xenon arc lamp	Crude oil	Synthetic	80-90 % PAHs	[4]
N-TiO_2	Immobilized on Spherical ceramic & thermocol beads	Solar light	Oil and grease spills	Synthetic	94 % & 100 % degradation with thermocol and ceramic respectively	[8]
N-TiO_2-PS & B/N-TiO_2-PS	Suspension	Visible light	Diesel oil	Synthetic	45 % & 48 % degradation with N-TiO_2-PS & B/N-TiO_2-PS respectively	[59]
Ni-N-TiO_2/pEGC Ni-N-TiO_2/EGC	Suspension	Visible light (500 W Xe lamp, λ = 420 nm with a cut off filter)	Diesel oil	Synthetic	95.9% & 63% diesel removal with Ni-N-TiO_2/pEGC & Ni-N-TiO_2/EGC respectively	[6]
Fe-TiO_2	Immobilized on polymethyl methacrylate plates	Visible light (3 W LEDs λ = 400-700 nm)	Total hydrocarbon	Synthetic & production water from skimmer inflow	98 % & 91.2 % degradation of Synthetic & production water respectively	[11]
TiO_2:N/TiO_2/rGO	Suspension	UV (125 W Hg lamp, λ = 365 nm)	Crude oil spills	Seawater	54.80% removal of alkanes & 74.83% removal of aromatic	[9]
Fe-TiO_2	Suspension	Solar light	Crude oil	Synthetic	-	[3]
TiO_2	Suspension	UV lamp (400 W)	Naphthalene toluene Benzene & phenol	Refinery wastewater	38 %, 39 %, 40 % and 42 % degradation of Naphthalene toluene Benzene & Phenol respectively	[60]
TiO_2	Suspension	UV (125 W high pressure Hg lamp)	Crude oil	Synthetic	-	[61]
TiO_2	Immobilized on Glass foam, glass fibre and steel grit	UV LED (8 W)	Diesel oil	Synthetic	68.31 %, 85.61 % & 55.96 % degradation with Glass foam, glass fibre & steel grit respectively	[62]
BiOI/TiO_2	Immobilized on FTO	Visible light (13 W LED with a cut off filter)	Crude oil	Synthetic	80.26 %	This work

Table S3. Summary of previous reports on $BiOI/TiO_2$ photocatalysts

Method	Compound	Illumination source	Activity	Reference
Electrospinning & Solvothermal	MB	500 W Xe lamp (> 420 nm)	$1.5x10^{-3}$ min^{-1}	24
Reverse microemulsion	MO	250 W halogen lamp (> 420 nm)	$14.5x10^{-3}$ min^{-1}	32
Electrospinning-solvothermal	MO	500 W halogen-tungsten lamp (> 420 nm)	$23.2x10^{-3}$ min^{-1}	23
Electrospinning & Hydrothermal	RhB	500 W Xe lamp (> 420 nm)	92 % degradation in 135 min	22
Electrospinning & Hydrothermal	MB	500 W Xe lamp (> 420 nm)	83 % degradation in 180 min	31
Doctor blading & SILAR	RhB	30 W white LED (> 420 nm)	$23.9x10^{-3}$ min^{-1}	14
Doctor blading & SILAR	4-CP	30 W white LED (> 420 nm)	$3.47\pm0.05x10^{-3}$ min-1	14
Chemical & solvothermal	Phenol	50 W halogen lamp ((> 420 nm)	3.7x10-3 min-1 in 315 min	30
Sonochemical	MO	500 W Xe lamp (> 420 nm)	33x10-3 min-1	33
Electrochemical	MO	300 W Xe lamp (> 420 nm)	99.89 % in 3 h	63
Hydrothermal	Cyclohexane	400 W metal halide lamp	-	64
Hydrothermal	Benzyl alcohol	400 W metal halide lamp	-	64
Doctor blading & SILAR	MO	13 W white LED (> 400 nm)	$13.94x10^{-3}$ min-1	This study
Doctor blading & SILAR	4-CP	13 W white LED (> 400 nm)	$2.68x10^{-3}$ min-1	This study
Doctor blading & SILAR	Crude oil	13 W white LED (> 400 nm)	80.26% in 48 h	This study
